# Full‐Control and Switching of Optical Fano Resonance by Continuum State Engineering

**DOI:** 10.1002/advs.202304310

**Published:** 2023-09-10

**Authors:** Joo Hwan Ko, Jin‐Hwi Park, Young Jin Yoo, Sehui Chang, Jiwon Kang, Aiguo Wu, Fang Yang, Sejeong Kim, Hae‐Gon Jeon, Young Min Song

**Affiliations:** ^1^ School of Electrical Engineering and Computer Science Gwangju Institute of Science and Technology Gwangju 61005 Republic of Korea; ^2^ Artificial Intelligence Graduate School Gwangju Institute of Science and Technology Gwangju 61005 Republic of Korea; ^3^ Department of Mechanical Engineering Massachusetts Institute of Technology Cambridge MA 02139 USA; ^4^ Cixi Institute of Biomedical Engineering International Cooperation Base of Biomedical Materials Technology and Application Chinese Academy of Sciences (CAS) Key Laboratory of Magnetic Materials and Devices Zhejiang Engineering Research Center for Biomedical Materials Ningbo Institute of Materials Technology and Engineering Chinese Academy of Sciences Ningbo Zhejiang 315201 China; ^5^ Advanced Energy Science and Technology Guangdong Laboratory Huizhou 516000 China; ^6^ Department of Electrical and Electronic Engineering University of Melbourne Parkville 3010 Australia

**Keywords:** Fano resonance, Fano state tuning, active color filters, bio‐sensors, inverse designs

## Abstract

Fano resonance, known for its unique asymmetric line shape, has gained significant attention in photonics, particularly in sensing applications. However, it remains difficult to achieve controllable Fano parameters with a simple geometric structure. Here, a novel approach of using a thin‐film optical Fano resonator with a porous layer to generate entire spectral shapes from quasi‐Lorentzian to Lorentzian to Fano is proposed and experimentally demonstrated. The glancing angle deposition technique is utilized to create a polarization‐dependent Fano resonator. By altering the linear polarization between s‐ and p‐polarization, a switchable Fano device between quasi‐Lorentz state and negative Fano state is demonstrated. This change in spectral shape is advantageous for detecting materials with a low‐refractive index. A bio‐particle sensing experiment is conducted that demonstrates an enhanced signal‐to‐noise ratio and prediction accuracy. Finally, the challenge of optimizing the film‐based Fano resonator due to intricate interplay among numerous parameters, including layer thicknesses, porosity, and materials selection, is addressed. The inverse design tool is developed based on a multilayer perceptron model that allows fast computation for all ranges of Fano parameters. The method provides improved accuracy of the mean validation factor (MVF = 0.07, *q*‐*q*') compared to the conventional exhaustive enumeration method (MVF = 0.37).

## Graphical abstract

An optical Fano resonator with a porous layer allows for full control of spectral shapes from quasi‐Lorentzian to Lorentzian to Fano. The resonator exhibits switchable behavior between quasi‐Lorentz and negative Fano states through polarization changes. Enhanced bio‐particle sensing capabilities are demonstrated, and an inverse design tool based on a multilayer perceptron model is developed to optimize Fano parameters efficiently.

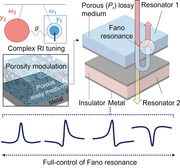

## 1 Introduction

Optical materials are frequently characterized by their absorption, transmission, and reflection spectra which typically exhibit resonant behavior. Resonances in nature, spanning acoustic, mechanical, and electromagnetic resonances, usually follow either a symmetrical spectrum with a Lorentzian line shape or an asymmetrical profile known as a Fano shape. The Fano resonance, arising from the coupling of two oscillators with different damping rates, has garnered significant interest in various research areas, including classical atomic systems and more recently, photonics.^[^ ^1^, ^2^, ^3^, ^4^ ^]^ Its unique asymmetric and steep line shape makes it particularly advantageous for applications in biosensing and chemical analysis.

The achievement of Fano resonance in photonics has thus far relied on the careful design of nanostructures for metasurfaces to fulfill the dedicated criteria for damping rates and coupling strength. Extensive investigations have been conducted on various metal‐based structures as well as all‐dielectric metasurfaces, unveiling numerous favorable Fano features, including high Q‐factor and tunability. Representative examples involve asymmetric split‐ring resonators,^[^ ^5^ ^]^ nanoclusters,^[^ ^6^, ^7^, ^8^, ^9^, ^10^ ^]^ photonic crystals,^[^ ^11^, ^12^ ^]^ gratings,^[^ ^13^, ^14^, ^15^, ^16^, ^17^ ^]^ and metamaterials.^[^ ^18^, ^19^, ^20^, ^21^ ^]^ While metasurfaces offer advantageous design freedom, their small feature sizes impose limitations on their practical applicability for large‐scale systems. Only recently has the thin‐film‐based structure been employed for optical Fano resonators, leading to simple fabrication and cost reduction, enabling prompt real‐world applications.^[^ ^3^, ^22^, ^23^ ^]^

Despite the breakthrough achieved by the film‐based optical Fano resonator, the simple geometry has presented challenges in achieving tunability in terms of spectral position, intensity, and spectral line shape. However, design flexibility in Fano resonance is crucial for various optoelectronic applications,^[^ ^13^ ^]^ such as lasing spacers,^[^ ^24^, ^25^ ^]^ sensors,^[^ ^26^, ^27^ ^]^ optical filters,^[^ ^28^ ^]^ and optical switches.^[^ ^29^ ^]^ The asymmetric spectral profile is quantified by the well‐established Fano relation and Fano parameter (*q*). The complete control of Fano resonance experimentally implies the ability to manipulate the Fano parameter from negative to zero to positive values. While such controllability has been demonstrated in metasurfaces, it has not yet been achieved in film structures.

In this work, we propose a thin film optical Fano resonator with a porous layer that enables precise and full control of Fano resonance by modulating the porosity. The simple layered structure facilitates easy and large‐scale fabrication, while a lossy medium with adjustable porosity allows for tunable damping rates. Through numerical calculations, we determined that the Fano parameter, *q*, continuously tuned from −4.6 to 0 to 4.1 in response to changes in porosity within amorphous silicon (a‐Si). We conducted additional tests on various lossy materials, including amorphous germanium (a‐Ge), Copper (Co), TiN, and Ge_2_Sb_2_Te_5_ (GST), to assess the *q* parameter variation. We experimentally observed four distinct spectral line shapes using the same film structure with different porosities. Furthermore, by creating a polarization‐dependent porous structure, we achieved switchable spectral line shapes between Fano and quasi‐Lorentz, which is the first demonstration of Fano switching in non‐metasurface devices. Also, by adding an extra layer to the Fano filter, we have developed the ability to detect bioparticles, even at the nano‐scale. This advancement induces a significant variation in the Fano state, enhancing our sensitivity to subtle analytes.

Furthermore, we experimentally demonstrated the application of the thin film device as an ideal substrate for a double imaging setup, requiring simultaneous reflection and transmission capabilities. By forming the extra layer on top of the Fano filter, we achieved a steep variation of *q*, enabling unconventional bioparticle detection. Finally, we extended our investigation to develop an AI tool for designing optical Fano resonators, effectively overcoming challenges associated with optimizing devices influenced by multiple parameters that affect the Fano parameter. We employed a multilayer perceptron (MLP) neural network model, which produces an accurate and fast design prediction. This approach holds the potential to unlock the full potential of Fano resonators across a broad range of photonic applications.

## 2 Results and Discussion

### 2.1 Fano Profile Control of a Weakly Coupled Oscillator via Porosity Modulation

**Figure 1:**
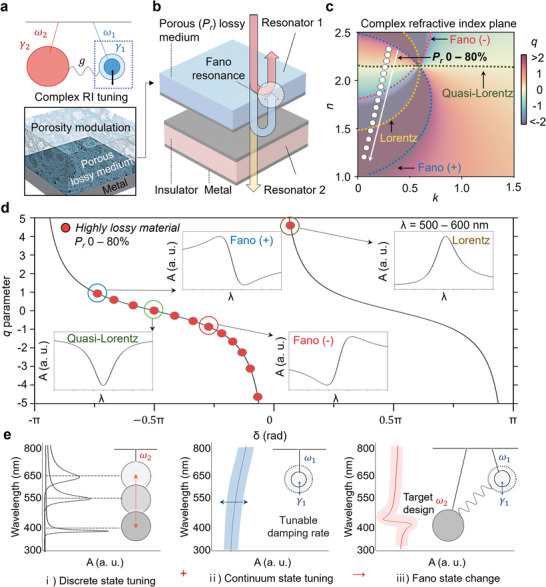
Controlling Fano parameters via continuum‐state tuning based on weakly coupled oscillator. a) Schematic of a continuum‐state tunable weakly coupled oscillator enabled by porosity modulation. b) Schematic illustration of a thin‐film Fano resonator comprising an ultrathin resonator and a Fabry‐Perot cavity for Resonator 1 and Resonator 2, respectively. c) Contour plot of the Fano parameter with complex refractive indices (i.e., n and k), and white scatterers represent complex refractive index variation through porosity tuning of highly lossy material (a‐Si). d) Mapping of the Fano parameter, *q*, to the phase difference, δ. Red scatterers represent porosity modulation ranging from 0% to 80%. The inset shows absorption spectra within the wavelength range of 500–600 nm. e) Absorption spectral profiles resulting from the coupling of oscillators 1 and 2 and their corresponding schematic models. Tuning the damping rate of the continuum state determines the coupled spectral line shapes.

**Figure** **1a** depicts a system consisting of two coupled oscillators, Oscillator 1 (blue circle) and Oscillator 2 (red circle), with the coupling strength (*g*) in between. The oscillators have their characteristic resonance frequencies, *ω_1_* and *ω_2_*, and damping rates, *γ_1_* and *γ_2_*, respectively. This mechanical analogy applies to explain various systems that involve interacting oscillating entities. Specifically, in the field of photonics, the concept of coupled oscillators describes the interaction between two coupled optical resonances.

The relative magnitude between *g* and *γ* plays a crucial role in explaining resonant phenomena for different coupling regimes. For example, in the weak coupling regime, the magnitude of the coupling strength is much smaller than the damping rate, i.e., |*g*| ≪ |*γ*|, and vice versa for the strong coupling regime.^[^ ^30^ ^]^ Hence, the ability to manipulate either *g* or *γ* enables us to precisely select desired resonant phenomena. In this work, we propose controlling the damping rate (*γ*) of one of the oscillators (referred to as Oscillator 1 in Figure 1a) by tuning the porosity of the medium, which induces variations in the complex refractive index. Figure 1b shows the structure used in this study to investigate the tunable Fano resonance is a thin‐film Fano resonator, which consists of a metal‐insulator‐metal (MIM) resonator as a weakly damped oscillator (Resonator 2) and a tunable porous layer as a strongly damped oscillator (Resonator 1). The coupled oscillator model for thin film Fano resonances was introduced in the previous study and utilized for numerical analysis in this work (1).

The theoretical prediction on the effect of porosity change on the Fano parameter (*q*) is calculated and visually represented in Figure 1c. Fano parameter (*q*) is introduced in the Fano formula, σ(*E*), which is described as follows:^[^ ^1^ ^]^

(1) $$\sigma \;\left(E\right)=\;D^{2}\frac{{\left(q+\;\Omega \right)}^{2}}{1+\Omega^{2}}$$

where *E* is the energy, *Ω* = 2(*E*−*E_0_*)/*Γ* where *Γ* is the resonance width and *E_0_* is the applied energy at the resonance frequency, *q* = cot(*δ*) where *δ* is the phase difference between the two oscillators. Figure 1c shows a 2D color map of Fano parameters as a function of the complex refractive indices, n and k. The range of values for n and k used in the plot is selected based on reasonable values typically observed in semiconductors. The *q* value distribution is non‐linear, which originates from the cyclic cotangent‐type dependence between *δ* and *q*. Adjusting the porosity (*P_r_*) of a lossy medium, in this case, amorphous silicon (a‐Si), from 0% to 80% varies the phase of Oscillator 1, leading to a change in *δ* (Note S1 and Figure S1, Supporting Information). The white dots in Figure 1c indicate complex refractive indices that are experimentally achievable with a‐Si with different porosities. Notably, the porosity variation allows a wide modulation range in the map covering various spectral line shapes, including Lorentz, quasi‐Lorenz, and Fano. The same result is now plotted in the *δ* and *q* space as shown in Figure 1d where red dots represent the achievable *δ* and *q* values, with the porosity modulation. The inset shows the spectral shape for four representative *q*‐parameters, demonstrating a quasi‐Lorentzian shape for *q* = 0, a Lorentzian shape for large *q*, and a Fano shape in between. The sign of the *q*‐parameter flips the spectral shape. Figure 1e illustrates how full control of the spectral shape is achieved by manipulating the two weakly coupled resonators. First, the target wavelength can be achieved by tuning the frequency of Resonator 2, while the damping rate control over Resonator 1 enables a *q*‐parameter sweep. Furthermore, our suggested design allows for intensity control of spectral response, providing an additional degree of freedom that is advantageous for many optics designs.

### 2.2 Demonstration of the Full‐Control and Switching of Optical Fano Resonance

**Figure 2:**
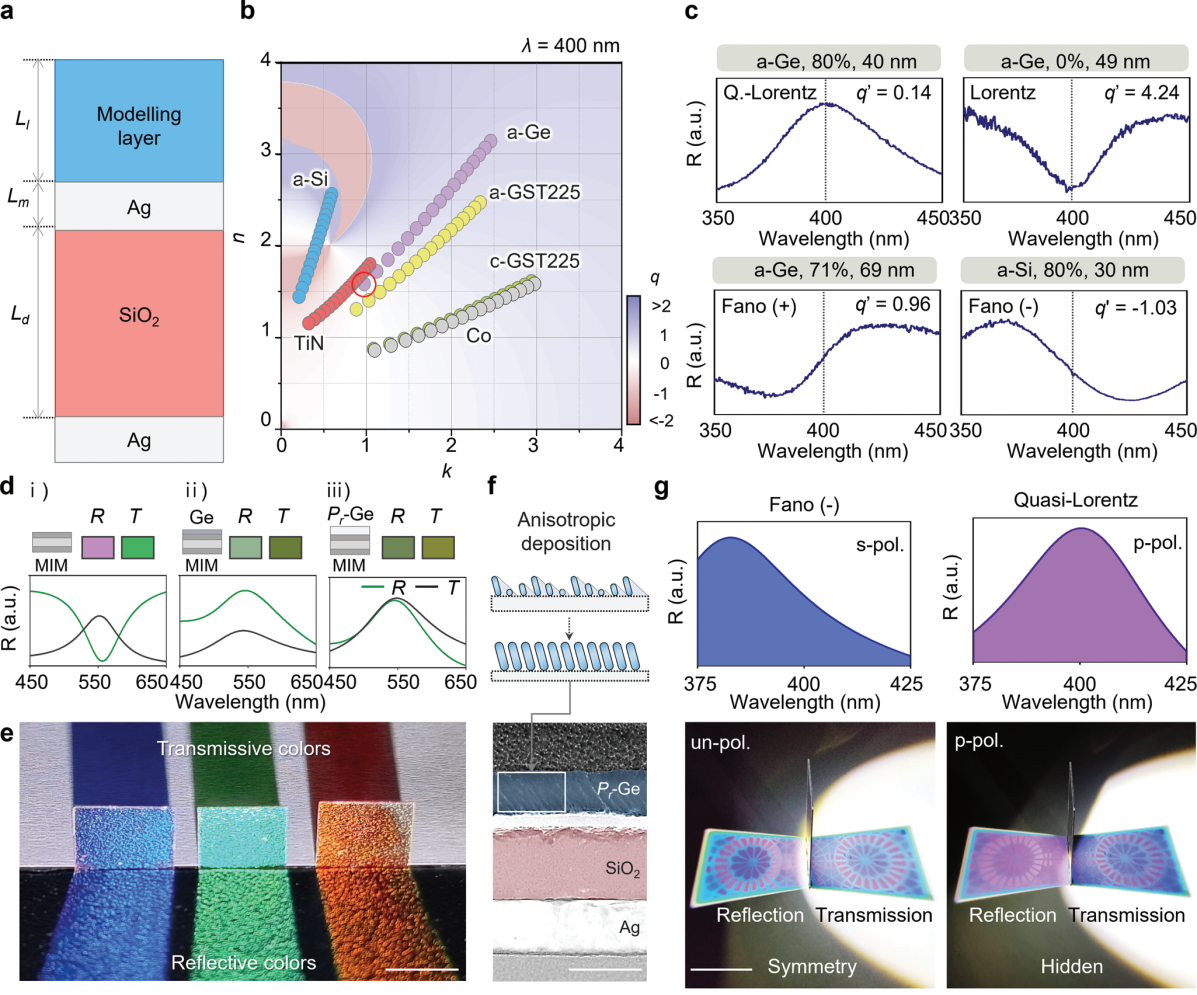
Full control of Fano resonance and switchable function. a) Thin‐film Fano resonator with a computational model, denoted as modeling layer. b) Complex refractive index space of modeling layer, swept from 0 to 4 at *λ_res_* = 400 nm and *L_l_* = 40 nm. Each scatter shows six representative lossy materials; a‐GST, c‐GST, Co, a‐Si, a‐Ge, and TiN with a range of *P_r_* from 0% to 80%. c) Reflectance spectra of four representative line shapes; quasi‐Lorentz, Lorentz, Fano (+), and Fano (‐) using a‐Ge and a‐Si. The *q*' represents validation Fano parameter. d) Calculated reflection and transmission spectra of i) MIM, ii) dense a‐Ge on MIM, and iii) *P_r_*‐Ge on MIM, respectively. Each palette shows reflection and transmission color. e) Photograph of Fano beam splitters that reflect and transmit the same color to the naked eye. Scale bar is 1 cm. f) Schematic of anisotropic deposition process, namely glancing angle deposition (GLAD) technique (top) and transmission electron microscope (TEM) image of cross‐section of fabricated sample (bottom). Scale bar is 100 nm. g) Reflection spectra of the Fano resonator under p‐polarization and s‐polarization, respectively (top). Photographs of the experimental demonstration of a switchable Fano resonator. The polarization state switches the reflected pattern, i.e., from symmetrical to asymmetrical patterns, which reveal and hide the shape of the pattern, respectively (bottom). Scale bar is 2 cm.

The cross‐sectional schematic illustration of the device is shown in **Figure** **2**a. The device consists of a Fabry‐Perot cavity formed by two highly reflective mirrors, i.e., Ag layers, and a lossy cavity formed by one Ag mirror and the other mirror attributed to the difference in refractive indices. We fixed the layer thicknesses to *L_m_* = 25 nm, *L_d_* = 84 nm, and *L_l_* = 40 nm and introduced material variation in the modeling layer to test the *q*‐parameter change with different materials. We introduced six representative lossy materials; a‐GST, c‐GST, Co, a‐Si, a‐Ge, and TiN. Figure 2b shows the complex refractive index variation of selected lossy materials, demonstrating their distinct coverage in the n‐k space. In this graph, the length does not represent a favorable characteristic, instead, the crucial aspect is that the line should span across *q*‐parameters ranging from negative to zero and then to positive values. For instance, Figure 2b highlights a specific condition close to a *q*‐parameter value of zero, denoted by the red circle, which exhibits a quasi‐Lorentzian spectral line shape. Additionally, other cases (e.g., Fano (+/−) and Lorentz state) also are presented based on different conditions (*L_l_*, *P_r_*, and lossy materials) by matching the complex refractive index of materials onto the target *q*‐parameter in Figure S2 (Supporting Information). In the next step, we prepared four different samples to experimentally demonstrate four representative line shapes as shown in Figure 2c. Each validation *q*‐parameter (*q*'), which is extracted from the reflectance spectra, presents similar values with respect to the target *q*‐parameter (Figure S3, Supporting Information). We used a‐Ge and a‐Si in the experiment due to their mature growth and fabrication technology. This thin‐film Fano resonance is known to possess the unique property of simultaneous resonances in both reflection and transmission spectra at the same wavelength, which is counterintuitive to conventional expectations. Figure 2d–i shows the typical reflection and transmission spectra, in this case obtained numerically from MIM structure. The peak position in the transmission, T (black line) coincides with the dip observed in the reflection, R (green line). The figure also displays the corresponding color of the reflected and transmitted light. The inclusion of a lossy layer results in the creation of an optical Fano resonator, allowing for the overlap of peak transmission and peak reflection, as shown in Figure 2d‐ii. A device that reflects and transmits the same wavelength can be utilized as a beam splitter. In addition, by incorporating a porous layer, the relative intensity can be finely adjusted, enabling the beam splitter with any desired R:T ratio. In Figure 2d‐iii, the spectra are numerically calculated to achieve a specific target of a 50:50 Fano beam splitter λ = 550 nm. This method can be extended to create a beam splitter at any wavelength by modifying the oscillation frequency of the weakly damped resonator. Experimentally, this can be accomplished by adjusting the thickness of the insulator in MIM structure. Figure 2e shows a photograph of Fano beam splitters operating at three different wavelengths, demonstrating nearly identical transmitted and reflected light to the naked eye.

We employed the glancing angle deposition (GLAD) technique in this work, which creates a thin film layer with tilted rod‐shaped nanostructures as shown in Figure 2f (Figure S4, Supporting Information).^[^ ^31^ *^–^* ^33^ ^]^ A schematic illustration (top) and transmission electron microscope (TEM) cross‐sectional image (bottom) are presented where a diagonal pattern in the Ge layer is evident. This geometry results in anisotropic responsivity, which enables us to control the *q*‐parameter by modifying the polarization state of the incident light.^[^ ^34^, ^35^ ^]^ We accounted for the polarization‐dependent porosity of the lossy material by applying the corresponding effective index in the TMM simulations, i.e., 70% porosity for p‐polarization, 80% porosity for s‐polarization, and 75% porosity for the un‐polarization (Figure S5, Supporting Information). Figure 2g shows the experimental demonstration of Fano resonance switching with respect to polarization. An anisotropic lossy medium generates Fano resonance with multiple states, resulting in a quasi‐Lorentz state for p‐polarization and a Fano (−) state for s‐polarization. This is the first switchable Fano resonance demonstration from a thin‐film‐based structure to the best of the author's knowledge. The fabricated structure provides control over symmetricity based on the polarization state, exhibiting symmetric color between transmitted and reflected light in polarization as shown in the photograph in Figure 2g. However, in s‐polarization, the reflected color is changed, causing asymmetric color. As a practical application, we propose a polarization‐sensitive bidirectional display, and the demonstration is included in the Supporting Information (Figures S6 and S7, Supporting Information). The pattern was intentionally designed based on the matching of the reflected p‐polarization color (purple) (Figure S8, Supporting Information). As an optical filter, it is crucial to consider that the Fano resonance relies on the symmetric/asymmetric absorption shape, which determines the line shape of each spectrum, causing optical loss. Among the three structures presented, the MIM/*P_r_*‐Ge exhibits the highest absorption rate compared to the other two (MIM and MIM/Ge) (Figure S9, Supporting Information). Nevertheless, we note that the visibility of the fabricated sample remains significant to distinguish each pattern and chromatic information.

### 2.3 Enhanced Sensitivity of Low‐Refractive Index Materials

In recent times, microscopic imaging holds promise for label‐free and amplification‐free quantitative diagnostics. However, the challenges arise due to the small size (≈100 nm in diameter) and low refractive index (≈1.5) of bioparticles, leading to difficulties in achieving precise estimations and increasing the limit of detection (LoD). To address this, it is crucial to enhance the light‐matter interaction and introduce significant variations in light intensity (transmittance and/or reflectance) to improve the signal‐to‐noise ratio.^[^ ^36^, ^37^ ^]^ In this regard, Fano resonance offers significant advantages in biosensing and imaging applications. This is primarily attributed to the fact that conventional intensity‐based sensing methods often rely on substantial resonant peak shifts to achieve detectable changes in intensity at specific wavelengths. However, with Fano resonance, it is possible to achieve significant intensity changes, or signal‐to‐noise ratios, without altering the resonant mode itself, but rather by modifying the spectral shape. This unique characteristic allows for improved sensitivity and signal detection, enhancing the performance and capabilities of biosensing and imaging technologies.

**Figure 3:**
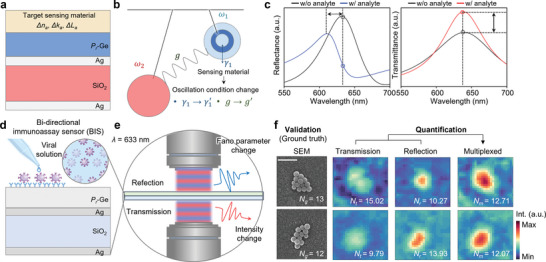
Bidirectional biosensor platform for quantifying bio‐particles. a) Schematic of Fano resonator with analyte layer, which has the variables refractive index, extinction coefficient, and thickness (i.e., *n_a_*, *k_a_*, and *L_a_*, respectively). b) Schematic of two coupled oscillators with variation of damping rate (*γ’*) and coupling constant (*g’*) by analyte layer. c) Reflectance and transmittance of Fano filter with and without analyte layer. d) Schematic of bidirectional biosensor comprising of viral particles on Fano filter. The attaching process is performed by a bidirectional immunoassay sensor (BIS). e) Schematic of dual‐imaging set‐up for viral nanoparticle detection. f) Scanning electron microscopy (SEM) image of viral particles (ground truth for validation) and monochromatic intensity map from reflected/transmitted/multiplexed images (right). Scale bar is 500 nm.

**Figure** **3**a presents a schematic illustration of the sensing mechanism involving a Fano resonator. The presence of a target‐detecting material on a lossy material (Ge) affects the effective refractive index, extinction coefficient, and thickness. In such a system, even materials with a low refractive index can impact the *q*‐parameter of the system, consequently influencing the spectral shape. The integration of analyte on Fano filter caused the damping rate of the discrete state to change from γ_1_ to γ_1_′, resulting in a variation in the coupling constant *g*′ (Figure 3b). We initially designed the Fano filter to have a *q*‐parameter of 0 in the absence of any sensing target material, so that any change in the upper layer would affect the *q*‐parameter and induce Fano resonance. Germanium layer with a porosity of 72% and a thickness of 38 nm is utilized. Simulation results for the reflection spectrum (Figure 3c, left) reveals that the sensing layer with a low refractive index (*n* = 1.5) and a thickness of 100 nm induces a phase shift of 0.25π, resulting in a transition from quasi‐Lorenz to Fano spectral shape. The same substrate can be utilized to measure changes in transmission intensity (Figure 3c, right). In the following, we showcased a label‐free bidirectional immunoassay sensor as schematically described in Figure 3d. Sensing viral nanoparticles (v‐NPs) is crucial and SARS‐CoV‐2 is chosen in this report as our focus. Clusters of v‐NPs with sub‐wavelength features are formed on the Fano filter with the Marangoni flow, and they are fixed on the surface with specific binding (Experimental Section and Figures S10 and S11, Supporting Information). Figure 3e shows the experimental set‐up for dual imaging with a light source at *λ* = 633 nm (Figure S12, Supporting Information). Experimentally obtained monochromatic optical microscope images in Figure 3f shows the transmitted and reflected optical images. The optical image acquired from reflection shows a distinct contrast between the point with and without nanoparticles, compared to the image obtained through transmission. The observation aligns with the prediction made in the simulation results presented in Figure 3c. In addition, we introduced the multiplexed method that incorporates both transmission and reflection data to achieve more accurate estimations. The quantification process was facilitated by utilizing the pre‐calculated weighting intensities of both transmission and reflection, which corresponded to the gap distance of v‐NPs (Figure S13, Supporting Information). For validation, we compared the quantified values with those measured from scanning electron microscopy (SEM) images. As depicted, the estimated number of v‐NPs from transmitted/reflected images represents sporadic errors, whereas the multiplexed image offers accurate counting (Figure S14, Supporting Information). We repeated the same test on 55 samples and verified that the multiplexed image showed accurate quantification results. The mean absolute error (MAE) of estimated number for multiplexed, reflected, and transmitted images was found to be 2.14, 2.32, and 3.41, respectively, indicating relatively more accurate prediction results in reflected and multiplexed images than in transmission images. Meanwhile, due to the diffraction limit (429 nm at λ = 633 nm) of optical microscopy in our setup, NP clusters with around three or four particles have faint visibility. Nonetheless, the MAE of estimated number for clusters with a few particles (3–6 particles) was improved signal‐to‐noise from Fano shape changes in the reflected/multiplexed spectrum. Consequently, the MAE values for multiplexed and reflection were 0.85 and 0.88, respectively, indicating relatively accurate detection capabilities, while for transmission, it was 1.31. Although there is innate optical loss in the Fano resonator, we note that the sensing ability shows reliable performance.

### 2.4 Inverse Design of Fano Resonator using Multilayer Perceptron

**Figure 4:**
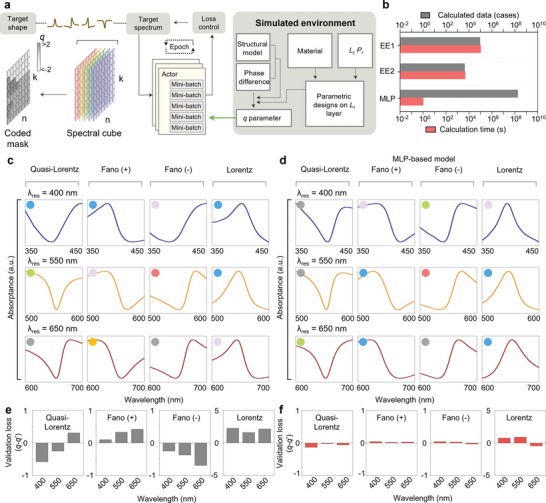
Spectral shape calculation and performance evaluation of MLP model. a) Computational modeling of Fano resonator composed of Resonators 1 and 2 and flow chart of MLP algorithm. b) Comparison of computation times for EE1 (resonator 1: six materials, thickness gap of 1 nm, porosity gap of 1%, resonator 2: thickness gap of 1 nm), EE2 (resonator 1: six materials, thickness gap of 5 nm, porosity gap of 5%, resonator 2: thickness gap of 5 nm) and MLP. Computation times for EE1, EE2, and MLP. c,d) Reflectance spectra obtained using c) EE2 and d) MLP model. Each circle is color‐coded to indicate the type of lossy material. e,f) Validation loss (*q*−*q*') of the spectra obtained using e) EE2 and f) MLP model.

The generation of diverse spectral line shapes using simple configurations is noteworthy. However, the design process is complex, involving intricate interdependencies that can lead to unpredictable outcomes and require significant computational resources. This complexity is primarily attributed to material dispersion, an expansive parameter space, and the non‐linear cyclic cotangent‐type dependence between the *q*‐parameter and design parameters. To address these challenges, an inverse‐design workflow is proposed in **Figure** **4**a (Figure S15, Supporting Information). It consists of three main steps: 1) computational modeling for numerical calculations of the n and k‐*q* map, 2) a machine learning process, and 3) determining the target spectral shape. To evaluate the efficiency of the approach, a shallow neural network with MLP was designed using a constructed dataset.^[^ ^38^ ^]^ The MLP model aimed to learn the relationship between structural parameters and the Fano parameter. Performance comparison was conducted with exhaustive enumeration (EE), varying material combinations, and structural parameters, as shown in Figure 4b. Two different conditions were considered: EE1, with 1% *P_r_* gap and 1 nm *L_l_* gap, and EE2, with 5% *P_r_* and 5 nm *L_l_*, at three target wavelengths using six lossy materials (see Experimental Section).

Figure 4c shows the reflectance spectra data set to obtain four cases of spectral line shapes (Lorentz, Fano (+/−), and quasi‐Lorentz at *λ_res_* = 400, 550, and 650 nm) using the calculation process of EE2 (Table S1, Supporting Information). Despite the potential for precise spectral control, the results are limited by the complexity and wide gap of the parameters involved in the design process, which can lead to unsatisfactory spectral shapes for the target. Figure 4d shows fast and precise calculation results of four spectral shapes at *λ_res_* = 400, 550, and 650 nm (Table S2, Supporting Information). Figure 4e,f illustrates the validation losses for the MLP‐based model and EE2 data set, respectively, which were obtained from the results shown in Figure 4c,d, respectively. During validation, the standard *q* parameters for Lorentz, Fano (−), Fano (+), and quasi‐Lorentz states are established as 0, −1, 1, and ±5, respectively. While the theoretical *q* parameter for the Lorentz state is infinite, a more efficient and straightforward calculation is achieved by constraining the *q* parameter in the range between +7 and −7. This range encompasses 90% of the pre‐dataset (Figure S16, Supporting Information). The final configuration and structural factors were determined by comparing the similarity between the target parameters *q* and *q*'. The validation loss for the MLP‐based model has a mean validation factor (MVF, *q−q*‘) of 0.07, compared to 0.37 of EE2, indicating that it has great accuracy. To ensure a fair comparison in the validation process, considering the mismatched ratio concerning the target parameter *q*, we normalized the *q* and *q*’ of Lorentz state by dividing them by 5. Also, we trained the MLP model three times to assess its consistency and define its effective range and boundary conditions, resulting in a reliable boundary for maintaining the spectral line shape with regard to the target spectrum. The defined boundaries for the target Fano parameter, *q*, are presented in Figure S17 (Supporting Information).

## 3 Conclusion

In conclusion, we proposed and demonstrated a thin‐film optical Fano resonator with a porous layer that facilitates precise and full control of Fano resonance. The simple layered structure facilitates easy and large‐scale fabrication, while the tunable porosity of a lossy medium allows for adjustable damping rates. By modulating the porosity within a‐Si, we demonstrated that the Fano parameter, *q*, can be continuously tuned from −4.6 to 0 to 4.1. We experimentally verified that the device is capable of generating four spectral line shapes: quasi‐Lorentzian, Lorentzian, positive Fano, and negative Fano. In addition, by creating a polarization‐dependent porous structure with GLAD, we achieved switchable Fano and quasi‐Lorentz spectral line shapes. This is the first demonstration of Fano switching in non‐metasurface devices. In addition, we designed the Fano resonator to have a large intensity variation when introduced to the analyte layer. Despite the low refractive index and small thickness of the analyte, it exhibited a change in *q‐*parameter from Fano (‐) to quasi‐Lorentz state, allowing for the detection of v‐NP (SARS‐CoV‐2, ≈*n* = 1.5, diameter = 100 nm). The overall sensing performance of the dual‐imaging system was improved with the Fano filter. dual‐imaging system was improved with the Fano filter. Moreover, by finely tuning the system's parameters, particularly setting *ω_1_* = *ω_2_* with *q* = 0, we can explore the fascinating possibility of observing double and multi‐Fano resonances through the photonic analog of electromagnetic‐induced transparency. This, in turn, will open up an exciting way for further research and manipulation of coupled resonance.

Finally, we introduced an efficient and durable inverse‐design flow for creating a universal spectral line shape by manipulating the continuum state based on expanded optical constants through porosity control in a single material. The MLP‐based design process provided feasible design solutions to overcome challenges posed by large parameter space, cotangent non‐linearity, and losses from theoretical approximations. Compared to the EE method, the MLP model showed fast computation (1.1303 s) with high accuracy (MVF = 0.07). Also, our MLP‐based model showed scalability on a large and complex data set, where EE can become computationally expensive and impractical. Our approach based on deep learning models could open up the design opportunities of a futuristic configuration of diverse two coupled oscillators.

## 4 Experimental Section

### Preparation of Pre‐Training Data Set

The complex refractive indices (*n* and *k* values) were measured (for a‐GST, c‐GST, a‐Ge, and a‐Si) and extracted from the literature (for Ag, SiO_2_, Co and, TiN).^[^ ^39^, ^40^, ^41^, ^42^ ^]^ The effective indices of lossy materials were calculated using MATLAB (MathWorks, USA) based on volume‐averaging theory.^[^ ^43^ ^]^ The 4D pre‐training and *n* and *k*–*q* data sets were calculated by MATLAB. For the weight factors of v‐NP sensing, the spectral shape was confirmed by rigorous coupled‐wave analysis calculation (DiffractMOD, RSoft Design Group, USA). The second order of diffracted light was considered, and a 0.2 nm grid was used. Since the Fano filter has planar structures, the conditions are sufficient to numerically stabilize the results.

### Fabrication of Bidirectional Fano Filters

The Fano filters were fabricated using an electron beam evaporator (KVE‐E2000, Korea Vacuum Tech, Ltd., Korea) under high vacuum (≈10^–6^ Torr) and plasma‐enhanced chemical vapor deposition (PECVD, System 100, Oxford, USA). The Ag layer was deposited at a rate of ≈1 Å s^−1^ up to the target thickness. The porous lossy layers were deposited by GLAD after being embedded on a customized slanted sample holder at a deposition angle of 70°. The SiO_2_ layer was deposited by PECVD using RF plasma. To control the *λ_res_*, we set *L_d_* to 84, 142, and 177 nm, corresponding to target wavelengths of *λ_res_* = 400, 550, and 650 nm, respectively.

### Optical Characterization

The reflection spectra of the fabricated structure were measured using a UV–VIS–NIR spectrometer (LAMBDA 950, Perkin Elmer, USA). The spectra were measured at a normal angle of incidence with a tungsten halogen lamp as the light source.

### Immunoassay of SARS‐CoV‐2 NPs

The NPs (100 nm diameter SiO_2_ particles, Sigma‐Aldrich, USA) and Fano filter were biofunctionalized with antigen (Anti‐Spike‐RBD‐hIgG1, InvivoGen, USA) and antibody (Spike‐RBD‐His, InvivoGen, USA), respectively. After synthesis, NPs were diluted in pre‐made phosphate‐buffered saline (PBS, Biosesang Co., Ltd., Korea) to obtain the desired concentration of 10 ng mL^−1^. Using a gas‐tight micro‐syringe (Legato 210, KD Scientific Inc., USA), a 300 nL volume solution was dropped onto the Fano resonator. The NPs were rotated in the evaporating droplet owing to the Marangoni flow. To block non‐specific binding and eliminate the impurities, the sample was rinsed with PBS solution for 30 s and deionized water for 10 s.

### Dual Imaging of Viral Particles

The Fano resonator with NPs was mounted onto the XYZ stage between the objective lenses. Optical bright‐field micrographs were captured using a 100× objective lens (MPlanFLN, Olympus, Japan) and a computer‐connected two CMOS camera (STC‐MCCM200U3V, OMRON SENTECH, Japan). The light was illuminated through a band‐pass filter (633 nm) under a white LED lamp. The diffraction limit of the dual imaging system was calculated with 429 nm, using the Rayleigh formula:

(2) $$\mathrm{diffraction}\;\mathrm{limit}=0.61\times \;\frac{\lambda }{NA}$$

The *λ* was the wavelength, which was set as 633 nm (i.e., the light source wavelength) and *NA* was the numerical aperture of the objective lens (i.e., 0.9). Also, to validate the accuracy of the quantification of bioparticle, the mean absolute error (MAE) was used:

(3) $$\mathrm{MAE}\;=\;\frac{\sum_{i\;=\;1}^{n}\left|y_{i}-x_{i}\right|}{n}$$

### MLP Calculation Method

To impose the non‐linear parameter space, a rectified linear unit was utilized as the activation function, except in the last layer, which generated the target design parameters. The output layer consisted of a fully connected (FC) layer equipped with a scaled sigmoid function and extracts the target resonator parameters. The network was trained for 100 epochs using the publicly available PyTorch framework with a batch size of 128 and a stochastic gradient descent (SGD) optimizer with a learning rate of 0.001. In SGD, a widely used optimization algorithm in machine learning for finding the best parameters, the model was updated to minimize the loss function after processing each sample or batch of data. Unlike gradient descent (GD), where the model parameters were updated after processing the entire training set, SGD randomly selected a single training example and updated the parameters based on the gradient of the loss function for that example. This made the algorithm faster and more scalable, particularly for large data sets. Mini‐batch SGD was utilized where, instead of a single training example, a small batch of examples was used to compute the gradient and update the parameters. This approach combined the benefits of both GD and SGD by reducing the variance of the updates while being computationally efficient. The learning process took 1 h and 14 min (i.e., 50 epochs) to reach the desired condition.

### Calculation Parameters of EE and MLPs

In the case of EE, the unit calculation required 1.13 s per structure for the spectrum generation using TMM simulation to obtain the *q* parameter (spectral shape) and validation parameter, *q*', at the target wavelengths (400, 550, and 650 nm). In the first condition of EE (i.e., EE1), the parameters were set as follows: the thickness gap of *L*_l_ was 1 nm and the *P*_r_ gap of Resonator 1 was 1% and six lossy materials were used (a‐GST, c‐GST, Co, a‐Si, a‐Ge, and TiN). EE1 took 103518 combinatorial explosions at three target wavelengths, requiring ≈32 h computation time. By reducing the combinatorial explosions (i.e., the thickness gaps of *L*_l_ was 5 nm and the *P*_r_ gap of Resonator 1 was 5% and six lossy materials were used), the second case of EE (i.e., EE2) took 1 h 26 min with 4590 combinatorial explosions. In contrast, the MLP‐based model took only 0.0003 s using the 205503678 pre‐training data set with complex refractive index space (i.e., *n* and *k* were between 0 and 4 with 0.01 gap and the thickness gaps of *L_l_* were 1 nm). Finally, the design of a target spectral line shape requires 1.1303 s, i.e., 4565 times faster than EE2).

### t‐SNE Characterization

The basic idea was to represent high‐dimensional data points in a lower‐dimensional space while preserving their pairwise similarities as much as possible. The algorithm achieved this by first computing a probability distribution over pairs of high‐dimensional data points, where similar pairs had a higher probability of being chosen. Then, a similar probability distribution was computed for the corresponding low‐dimensional representation of the data points. Subsequently, the algorithm minimized the difference between these two probability distributions using GD optimization (Figure S18, Supporting Information).

### Statistical Analysis

The results were expressed as mean ± standard deviation (SD) and examined using a one‐way analysis of variance along with Tukey's post hoc tests. Statistical significance was indicated as follows: NS (not significant, *p* > 0.05), * (*p* < 0.05), ** (*p* < 0.01), and *** (*p* < 0.001).

## Conflict of Interest

The authors declare no conflict of interest

## Author Contributions

J.H.K., J.H.P., and Y.J.Y. contributed equally to this work. J.H.K., J.H.P., Y.J.Y., H.‐G.J., and Y.M.S. conceived the idea and designed the whole experiment. J.H.K. and J.K. developed the process and fabricated the samples. J.‐H.P. developed the machine learning model and conducted the analysis. J.H.K., J.‐H.P., Y.J.Y., J.K., H.‐G.J., and Y.M.S. conceived the experiments and analyzed the data. J.H.K., J.‐H.P., H.‐G.J., S.K, and Y.M.S. mainly wrote the manuscript. J.H.K., J.‐H.P., Y.J.Y., S.C., J.K., A.W., F.Y., H.‐G.J., S.K and Y.M.S. edited the manuscript. All the authors confirmed the final manuscript. Y.M.S. guided the entire project.

## Supporting information

Supporting Information

## Data Availability

The data that support the findings of this study are available from the corresponding author upon reasonable request.
