# Supplementary material for: Full‐Control and Switching of Optical Fano Resonance by Continuum State Engineering
Source: Adv Sci (Weinh). 2023 Sep 10;10(32):2304310. doi: 10.1002/advs.202304310 (PMC10646235; doi:10.1002/advs.202304310)
Supplement: Supplementary file 1 — Supporting Information [file ADVS-10-2304310-s001.pdf]

ADVANCED SCIENCE
Open Access

Supporting Information

for *Adv. Sci.*, DOI 10.1002/advs.202304310

Full-Control and Switching of Optical Fano Resonance by Continuum State Engineering

Joo Hwan Ko, Jin-Hwi Park, Young Jin Yoo, Sehui Chang, Jiwon Kang, Aiguo Wu, Fang Yang, Sejeong Kim, Hae-Gon Jeon and Young Min Song**

Copyright WILEY-VCH Verlag GmbH & Co. KGaA, 69469 Weinheim, Germany, 2021.

Supporting Information

Full-control and switching of optical Fano resonance by continuum state engineering

Joo Hwan Ko†, Jin-Hwi Park†, Young Jin Yoo†, Sehui Chang, Jiwon Kang, Aiguo Wu, Fang Yang, Sejeong Kim, Hae-Gon Jeon & Young Min Song**

Supplementary Note 1. Thin-film Fano resonator and equivalent pendulum oscillator model.

The weakly coupled resonator, which makes a Fano resonance, is manifested in the absorption spectrum, σ(*E*), calculated by the Fano formula:[1]

$$\sigma(E) = D^2 \frac{(q+\Omega)^2}{1+\Omega^2},$$

where *E* is the energy and $\Omega = 2(E - E_0)/\Gamma$ (Γ is the resonance width, E_0 is the energy at the resonance frequency and $q = \cot(\delta)$, where δ is the phase difference between the two modes).

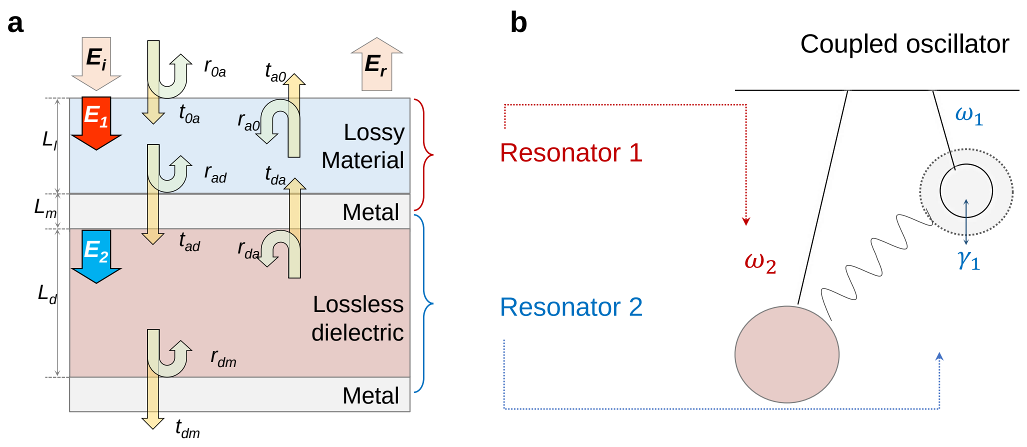

Figure N1. Fano resonator and pendulum oscillator model. a) Thin-film Fano resonator comprised of resonator 1 (ultrathin resonator, i.e., lossy film on metal reflector) and resonator 2 (Fabry–Perot cavity, i.e., metal/insulator/metal (MIM) structure). The variables are E_i and E_r are the total field injected into resonator 1 and the reflected field from the Fano resonator, respectively; L_l, L_m, and L_d are the thickness of absorbing layer (lossy layer), thin metal film and dielectric layer, respectively; the subscripts of reflection and transmission coefficients designate the medium (i.e., 0, l, d, and m represent air, lossy layer, dielectric, and metal layer, respectively). b) Coupled pendulum model with forced oscillation representing Fano resonance, where γ_1 and γ_2 are the damping rates and ω_1 and ω_2 the resonant frequencies of resonators 1 and 2, respectively. As L_m becomes large (approaching infinity), the transmission coefficients across the spacer (t_{la} and t_{ld}) vanish, resulting in zero coupling terms. In this situation, the system behaves as two uncoupled oscillators.[2] However, for finite L_m, all the necessary conditions for

Fano resonance are met, with a strongly damped, driven oscillator (Resonator 1) weakly coupled to a less damped oscillator (Resonator 2). Therefore, we chose a suitable thickness of 25 nm that fulfills these conditions.

As shown in Figure N1, the damping rate tuning of resonator 1 affects the whole system in terms of oscillator intensities $|A_k(\omega)|^2$ and q, where A_k is the intensity ratio of the field of the kth resonator (E_k) with regard to the input field into the kth resonator ($E^i{}_k$), $A_k = (E_k/E^i{}_k)^2$. By putting the parameters, these relations are expressed in terms of E_1 and E_2 as follows:[2]

$$\begin{pmatrix} 1/A_1\,(\omega) & -r_{dm}t_{dl}r_{l0}e^{i[2\phi_d(\omega)+2\phi_l(\omega)]} \\ -t_{ad}e^{i\phi_l(\omega)} & 1/A_2\,(\omega) \end{pmatrix}\begin{pmatrix} E_1 \\ E_2 \end{pmatrix} = i\begin{pmatrix} t_{0l}E_i \\ 0 \end{pmatrix},$$

where

$$r_{ij} = \frac{n_i - n_j}{n_i + n_j}, t_{ij} = \frac{2n_i}{n_i + n_j}.$$

The total injected field is expressed as

$$\frac{E_2}{E_{2i}} = \frac{1}{1 - r_{dm}\tilde{r}_{dl}e^{2i(\Phi_d(\omega))+\Phi_{dm}}} = A_2(\omega).$$

By using the relation of $\tilde{r}_{dl} \sim r_{dm}$, $A_2(\omega)$ is defined as

$$|A_2(\omega)|^2 = \frac{c^2}{4n_d^2L_d^2|r_{dm}|^2}\left(\frac{1}{\gamma_2^2 + (\omega - \omega_2)^2}\right),$$

where

$$\gamma_2 = \frac{c(1 - |r_{dm}|)}{2n_lL_d|r_{dm}|^{1/2}} \approx \frac{2cn_m}{L_d[n_d^2 + k_m^2]}.$$

For resonator 1 (ultrathin resonator), there is no exact analytical expression for the resonance frequency ω_1. Nevertheless, it can be approximated under the assumption that $Im(n_l)$ is typically

smaller than $Re(n_l)$. Thus, the resonant frequency can be approximated by the following condition:[2]

$$2Re[\emptyset_l(\omega_1)] \approx \emptyset_{l0} - \emptyset_{lm} + 2k\pi,$$

where $\emptyset$ is oscillator phase.

In case of $A_1(\omega)$, the injected field is expressed as

$$\frac{E_1}{E_{1i}} = \frac{1}{1 - r_{l0}\tilde{r}_{lm}e^{2i(\omega)}} = A_1(\omega).$$

Assuming that n_l^{Im} is generally smaller than n_l^{Re}, the approximation gives the oscillator intensity as

$$|A_1(\omega)|^2 = \frac{c^2 e^{2n_{lossy}^{Im}} L_{lossy}\omega_1/c}{4(n_l^{Re})^2 L_l^2 |r_{am}r_{a0}|}\left(\frac{1}{\gamma_1^2 + (\omega - \omega_1)^2}\right),$$

where

$$\gamma_1 = \frac{ce^{k_{lossy}L_{lossy}\omega/c}\left(1 - e^{-2e^{k_a L_a \omega/c}}|r_{a0}r_{am}|\right)}{2n_l L_l |r_{a0}r_{am}|^{1/2}}.$$

The subscripts represent the layers (*a* indicates the absorbing layer, *m* the metal layer and *d* the dielectric layer). ϕ and ω represent the phase and frequency, respectively. As a strategical approach for universal realization, we considered the effective complex refractive index using a porous medium based on volume-averaging theory. Then, we derived the damping rate corresponding to the porous medium of the lossy layer of resonator 1 as follows:

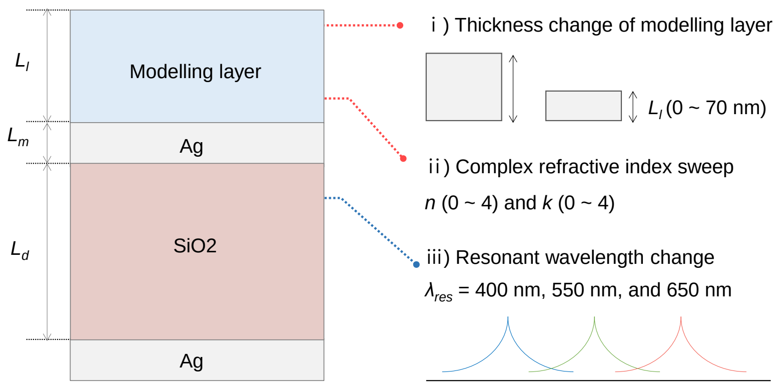

Figure N2. Computational model and structural parameters. Fano resonator comprised of resonator 1 (ultrathin resonator, i.e., modelling layer on metal reflector) and resonator 2 (Fabry–Perot cavity, i.e., MIM structure). Control of structure parameters i) thickness of modelling layer, ii) complex refractive index, and iii) resonant wavelength.

As described in Figure N2, we introduced a computational model for confirming the parameter q corresponding to structure parameters: i) thickness of modelling layer (0–70 nm), ii) refractive index (n = 0–4), extinction coefficient (k = 0–4), and iii) resonant wavelength (λ_{res} = 400, 550, and 650 nm). As described in Figures N3–N5, the parameter q contours show non-linear tendency with respect to complex refractive index.

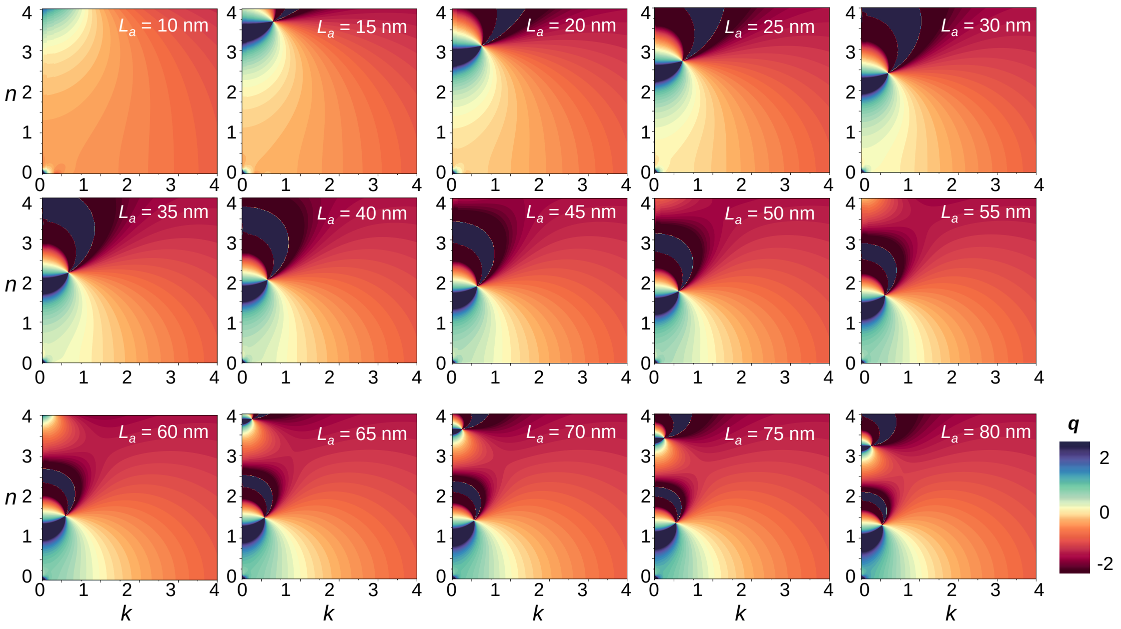

Figure N3. Contour maps of parameter q corresponding to complex refractive index variation (i.e., refractive index (n = 0–4) and extinction coefficient (k = 0–4)) for each thickness of lossy layer, L_l, at the wavelength λ_{res} = 400 nm.

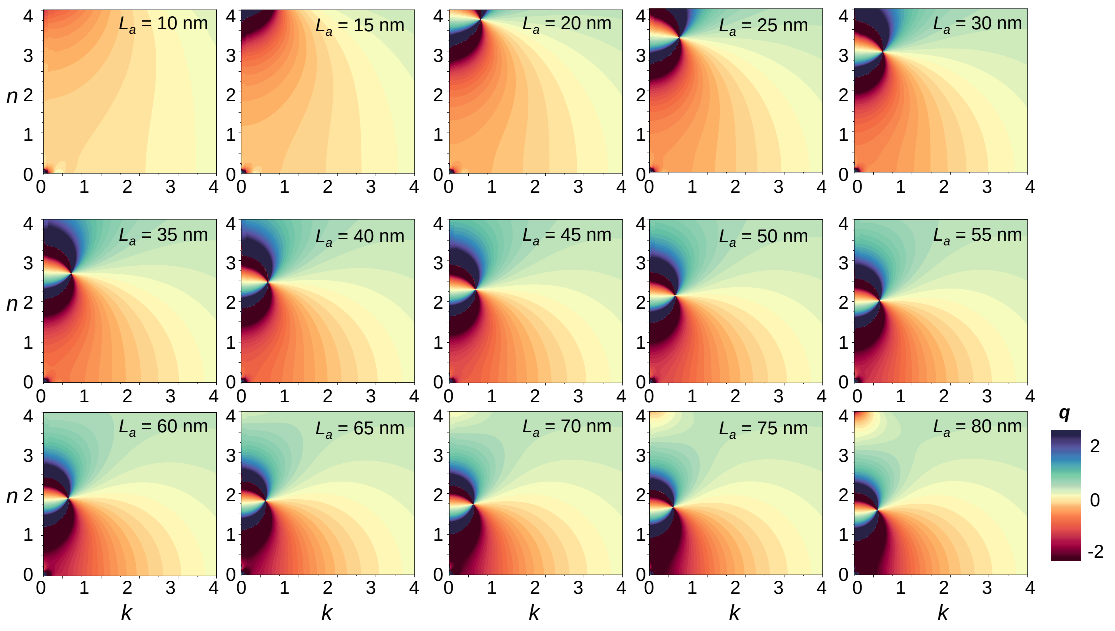

Figure N4. Contour maps of parameter q corresponding to complex refractive index variation (i.e., refractive index (n = 0–4) and extinction coefficient (k = 0–4)) for each thickness of lossy layer, L_l, at the wavelength λ_{res} = 550 nm.

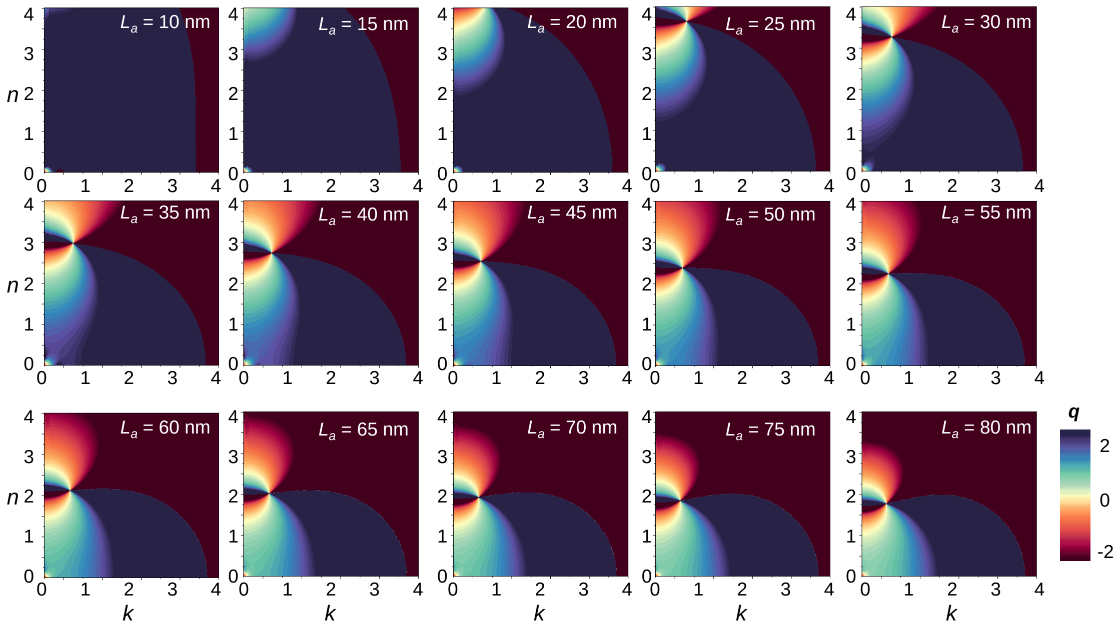

Figure N5. Contour maps of parameter q corresponding to complex refractive index variation (i.e., refractive index (n = 0–4) and extinction coefficient (k = 0–4)) for each thickness of lossy layer, L_l, at the wavelength λ_{res} = 650 nm.

To achieve the desired spectral shape, the target q parameter should be addressed, which is represented in Figures N3–N5. However, the desired configuration with matched complex refractive index is difficult to realize with limited conventional materials. Therefore, we tailored the effective refractive index through porosity change as follows:[3]

$$n_a^2 = \frac{1}{2}\left[A + \sqrt{A^2 + B^2}\right],$$

$$k_a^2 = \frac{1}{2}\left[-A + \sqrt{A^2 + B^2}\right],$$

where

$$A = \varepsilon_{r,eff} = P_r + (1 - P_r)(n_l^2 - k_l^2),$$

$$B = \frac{\lambda \sigma_{eff}}{2\pi c_0 \varepsilon_0} = 2P_r + 2n_l k_l (1 - P_r). \tag{8}$$

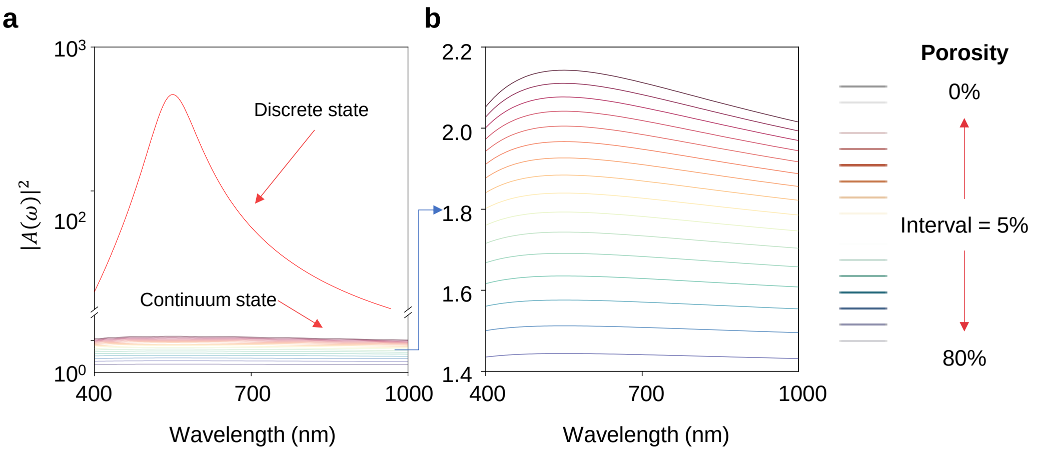

Figure N6. Damping factor and intensity with porosity. a) Oscillator intensities for MIM layer and ultrathin resonator corresponding to various porosities of a-Si layer. b) Closed view of oscillator intensities of continuum state.

For the derivation of the relationship in calculation process, we selected a-Si as the lossy layer. Consequently, we controlled the damping factor and oscillator intensities corresponding to the porosity change (Figure N6).

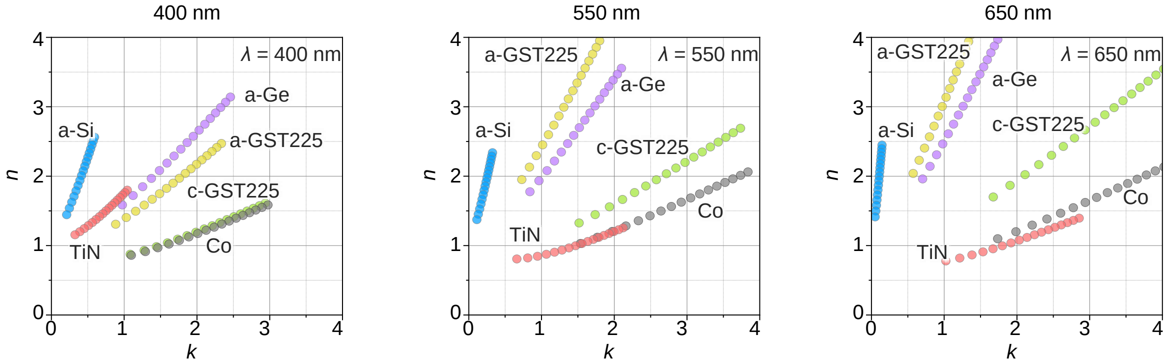

Figure N7. Complex refractive indices of lossy materials. Representative lossy materials and their complex refractive indices with the porosity, P_r, changing from 0% to 80% at the three representative wavelengths. In the calculations, we used a complex refractive index from the literature (Ag, SiO_2, Co, and TiN)[4–7] and measured data (a-Ge, a-Si, c-GST, and a-GST).

Figure N7 shows the effective complex refractive indices of representative lossy materials (i.e., a-GST, c-GST, a-Si, a-Ge, Co, and TiN) with a changing porosity. Hence, the cover range of complex refractive indices is enlarged.

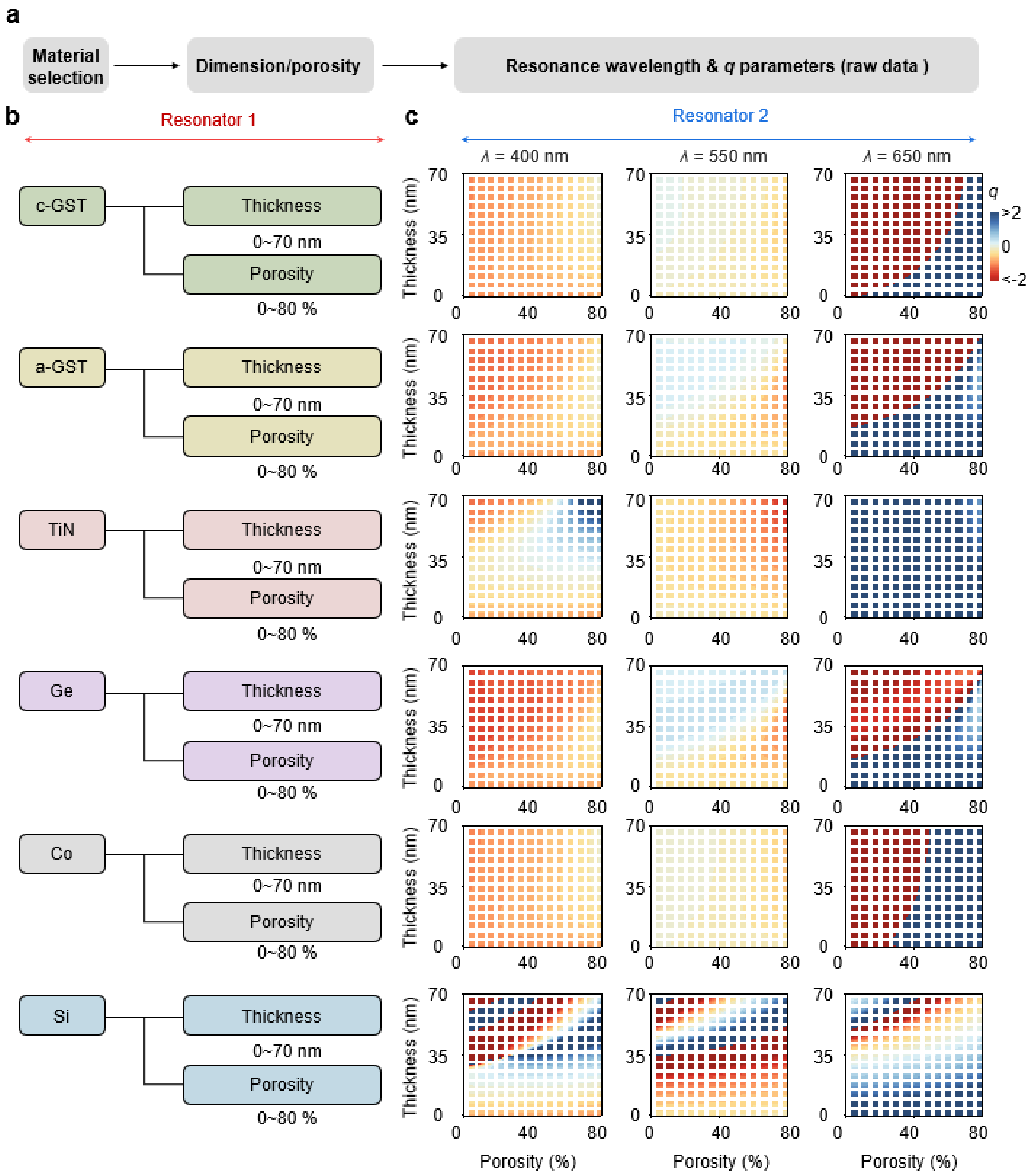

Figure S1. Calculation process of *q* parameter with varying material combination, dimension and complex refractive index. a) Overall design process and *q* parameter extraction. b) Material, porosity and dimension tuning of resonator 1. c) Calculated *q* parameter corresponding to P_r and L_l at three resonance wavelengths.

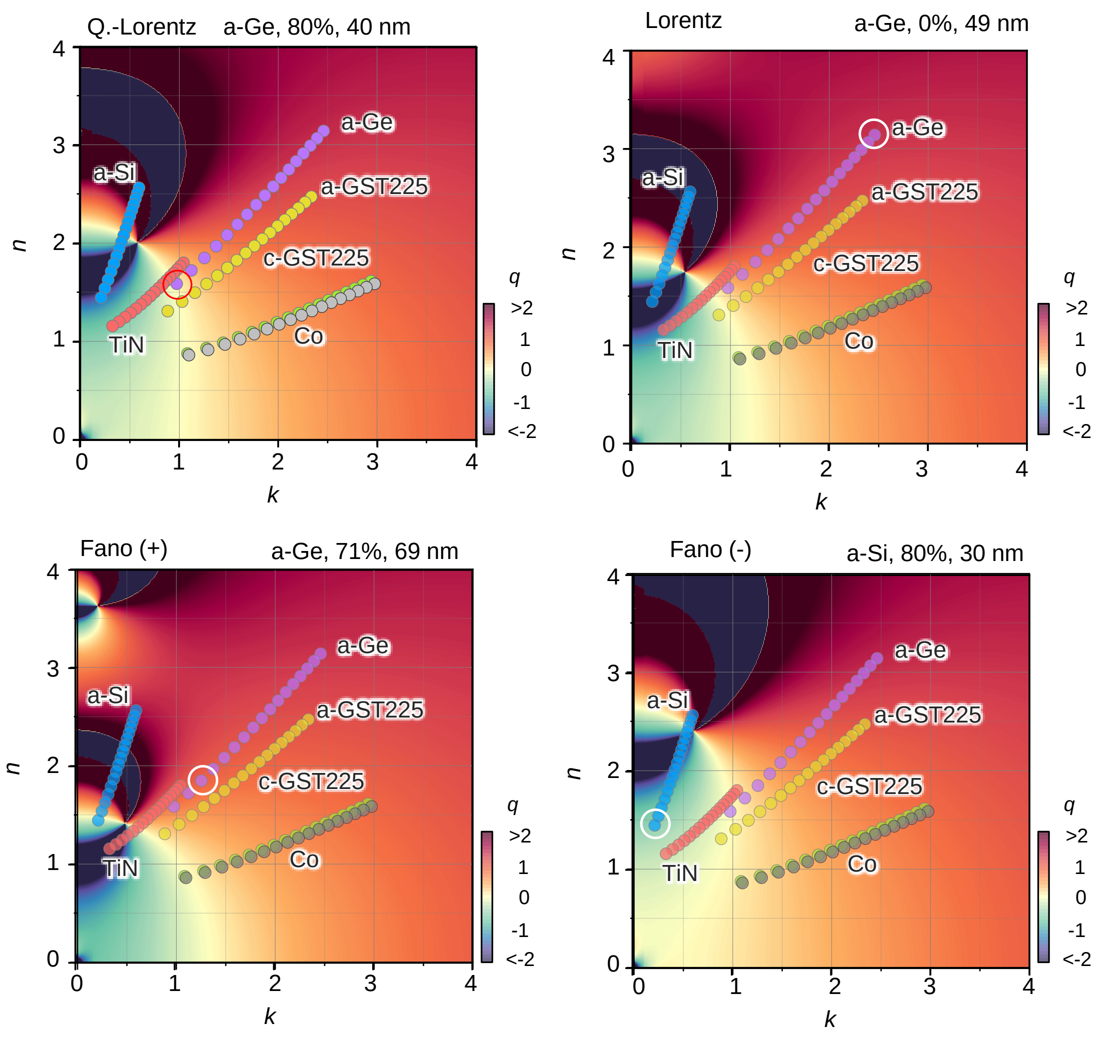

Figure S2. Complex refractive index of six lossy materials n and k – *q* map. Complex refractive index of six lossy materials and n and k – *q* map. Each circle represents target design.

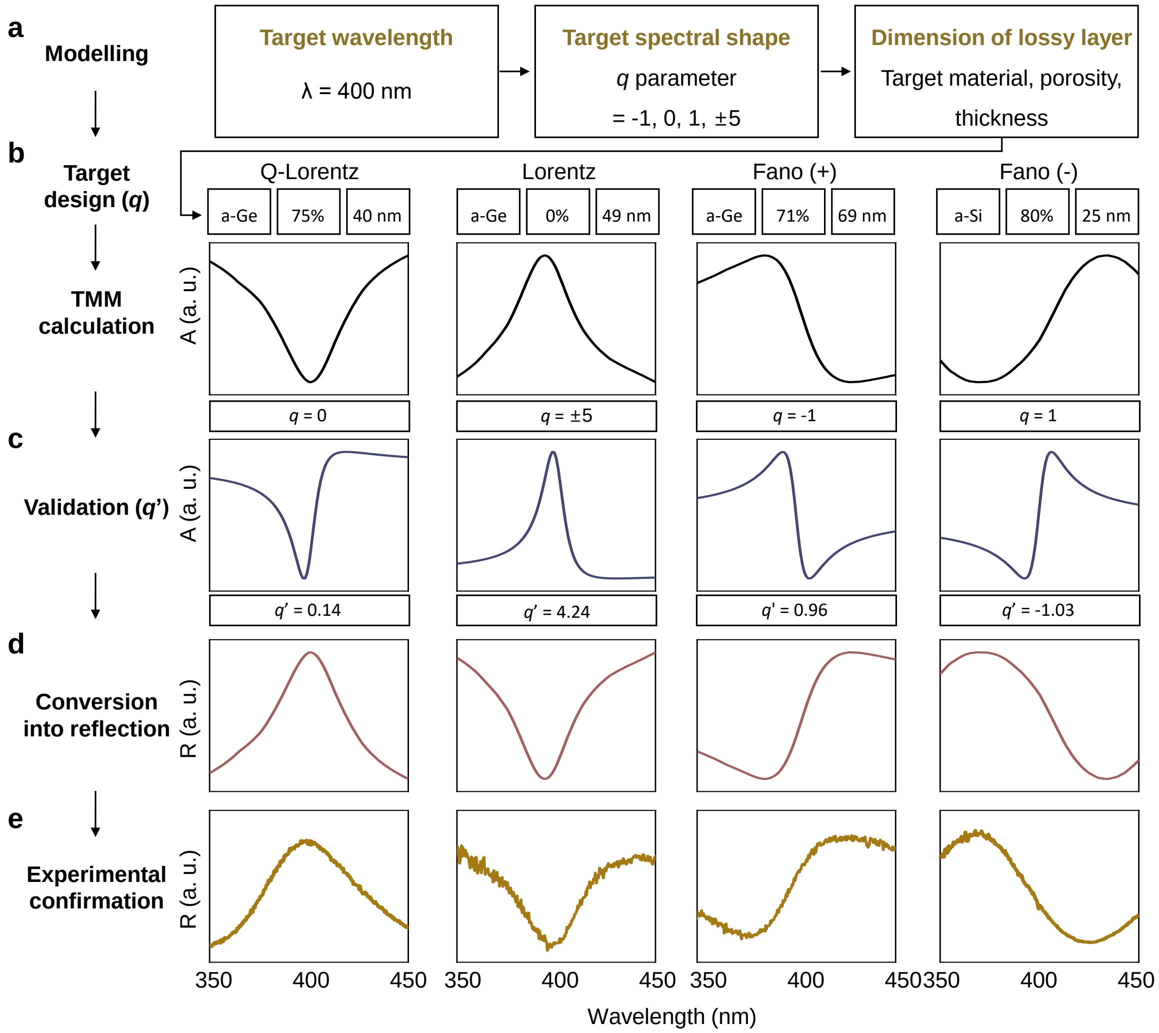

Figure S3. Procedure of designing and validating four different spectral line shapes. a) Modelling process of four spectral line shapes at the target wavelength 400 nm. b) Absorption spectra of each shape calculated by TMM with designed parameters and materials. c) Evaluation of spectral line shape with validation *q* parameter, *q*'. d) Reflectance spectra of designed parameters. e) Measured spectra of designed structures realized.

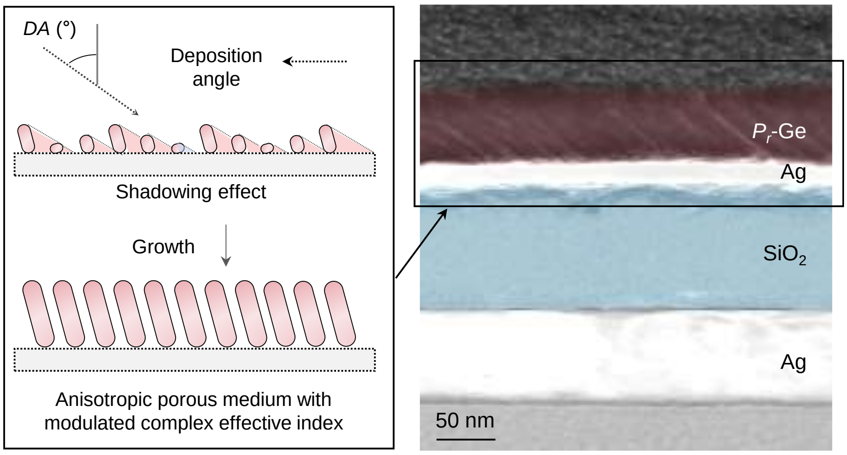

Figure S4. Fabrication of porous/anisotropic layer. Schematic of fabrication process via glancing angle deposition method (left). Cross-sectional transmission electron microscopy image of Fano filter with porous a-Ge layer (right).

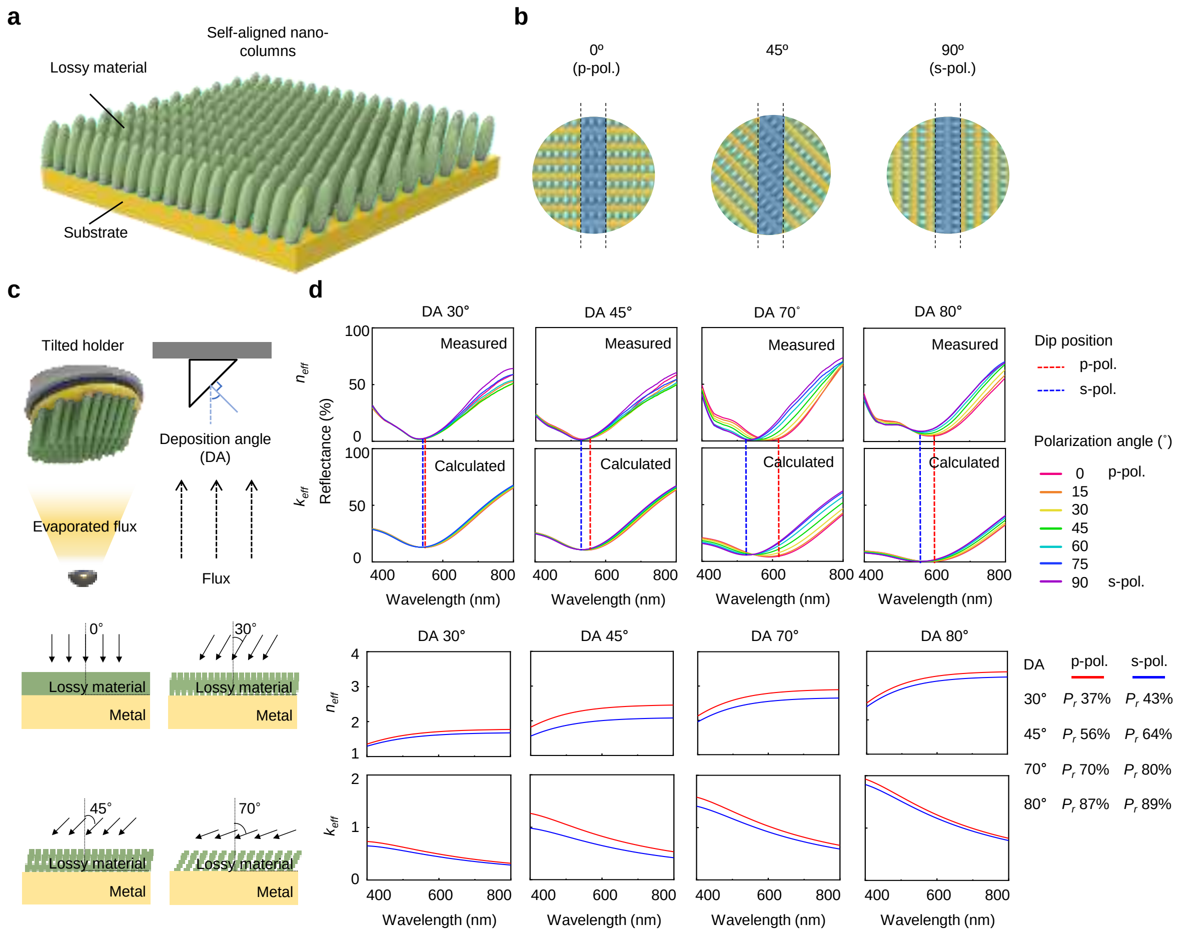

Figure S5. a) Schematic of self-aligned nano-columns with lossy material. b) Conceptual schematic image of top view of nanocolumns corresponding to the polarization angle. c) Schematic of self-aligned nano-columns with deposition angle. d) Measured/simulated reflectance and measured effective index.

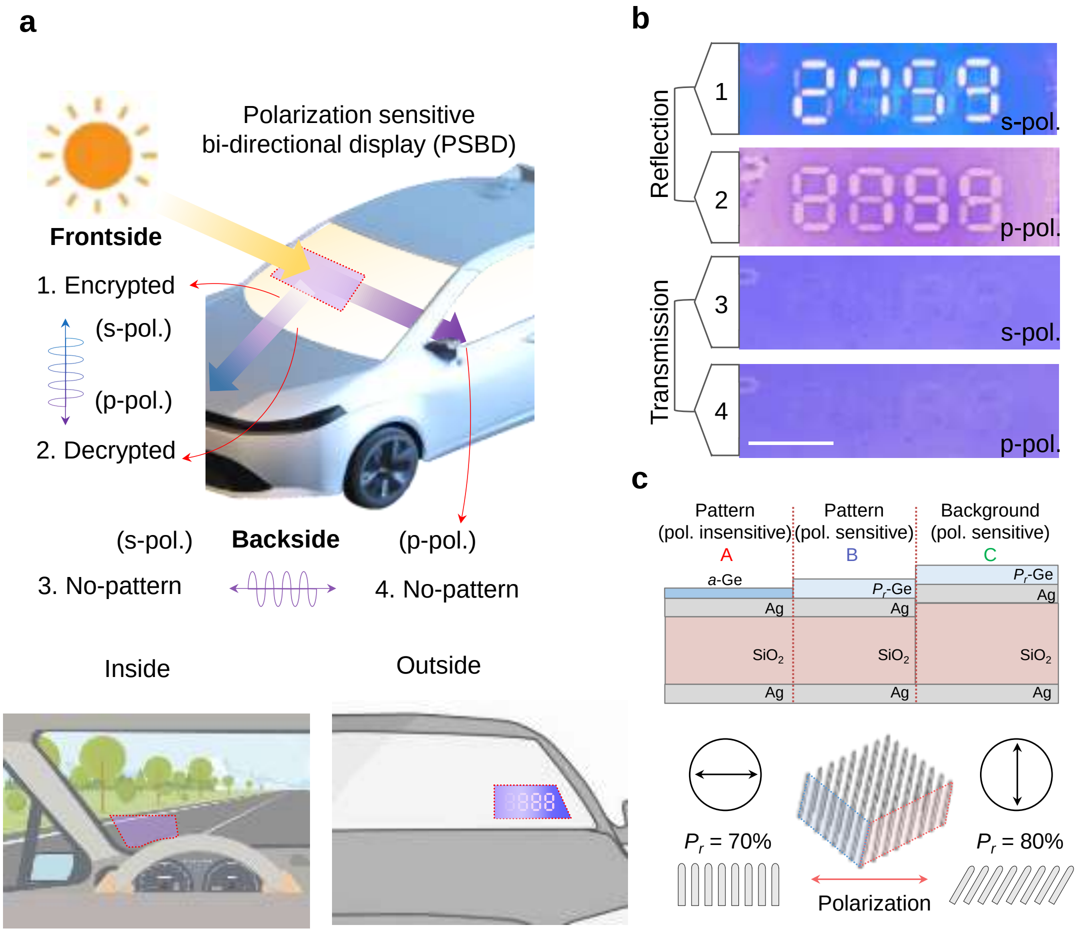

Figure S6. Design and operation principle of polarization-sensitive bidirectional display (PSBD). a) Schematic illustration of PSBD, which shows bi-directional property. When observer view the PSBD from an external perspective, the pattern is revealed, and conversely, the pattern is hidden when viewing it from inside of car. b) Photographs of Fano filter. For contrast with the original photographs, a customized color map was applied. c) Each section is distinguished by the function. Lossy layer of section a is dense a-Ge, which has isotropic medium (polarization insensitive), on the other hand, section b and c has anisotropic medium, enabling polarization sensitive operation. Section A and B are participated in pattern area and C is background area. Schematic illustration of self-aligned nanocolumns and variation of P_r of effective medium corresponding to polarization state.

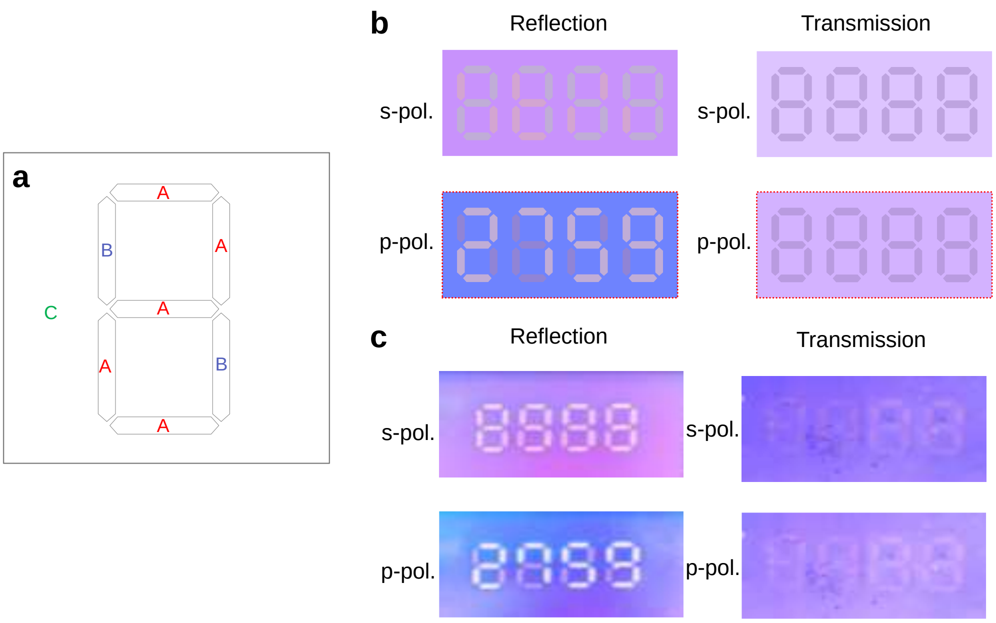

Figure S7. Design and experimental result of PSBD. a) Spatial placement of sections A, B, and C. b) Simulation result of four states of PSBD. c) Original photographs of experimental result of four states of PSBD.

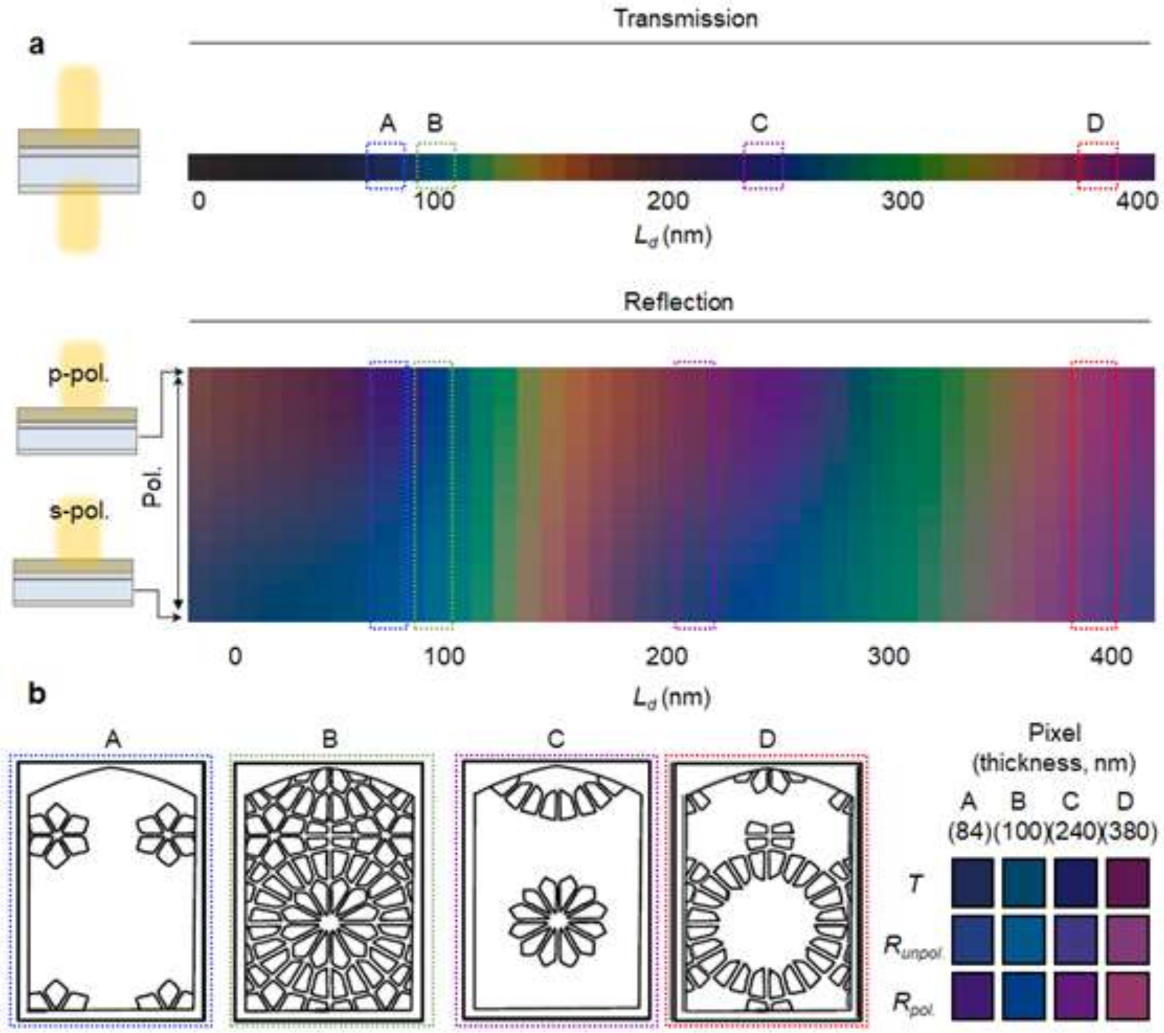

Figure S8. a) Schematic representation of transmission and reflection from the Fano resonator, illustrating color palettes with varying L_d from 0 to 400 nm. The reflected color also exhibits polarization-sensitive variations. b) Pattern mask and its corresponding pixels used in the experiment. We note that the reflected color of pixels A, C, and D was matched under the polarized light.

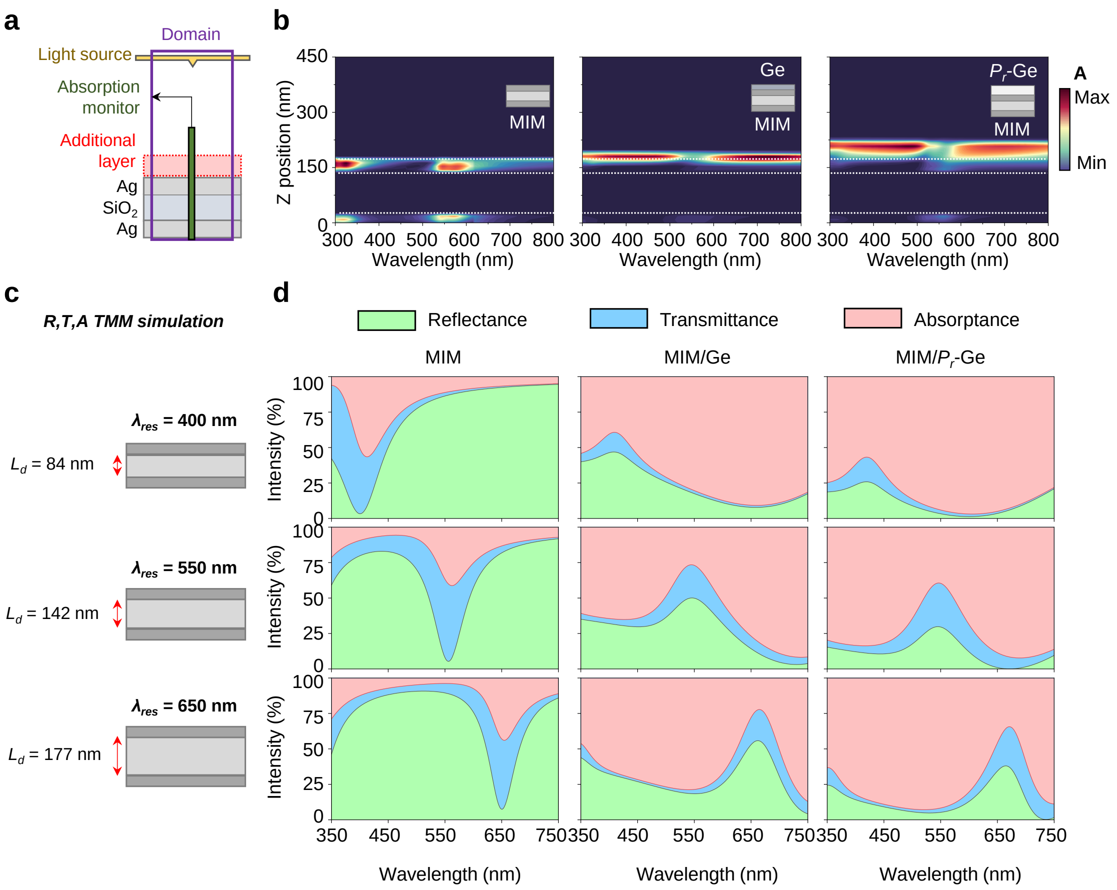

Figure S9. a) RCWA model with a monitor for measuring absorption. b) Absorption profiles of each structure (MIM, MIM/Ge, and MIM/P_r-Ge) corresponding to changes in wavelength. c) TMM simulation model of each structure. d) Reflectance, transmittance, and absorptance of each structure. The integrated absorptance of MIM/P_r-Ge reached the highest level at 75%, whereas MIM/Ge showed 63% and MIM exhibited 25% (wavelength range, λ = 350 – 750 nm) for λ_{res} = 550 nm.

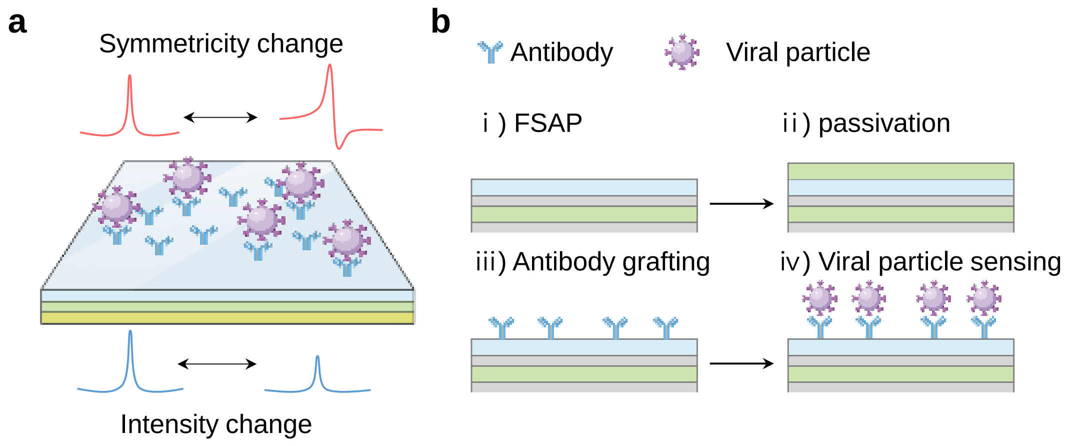

Figure S10. Fabrication/immunoassay process of bidirectional sensor. a) Change of reflection and transmission spectral shapes by attaching virus clusters on Fano filter. b) Surface functionalization by grafting SARS-CoV-2 antibody and sensing process.

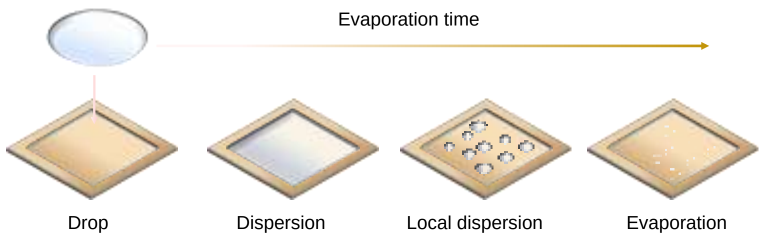

Figure S11. Marangoni flow of droplets. Schematic images show the sequential process of vortexing a dispersed droplet, resulting in Marangoni flow. Circular vortexing of the solution leads to a reduction in the solution layer thickness and formation of a cluster owing to the resonance wavelength shift at the center.

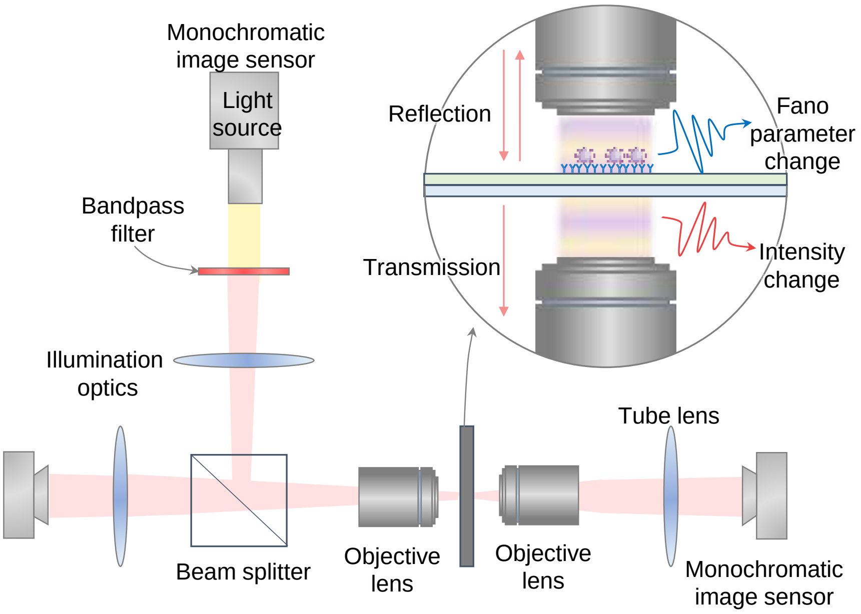

Figure S12. Dual-imaging set-up and optical components. Schematic of dual-imaging set-up for viral particle detection and close-up of objective lens and virus clusters.

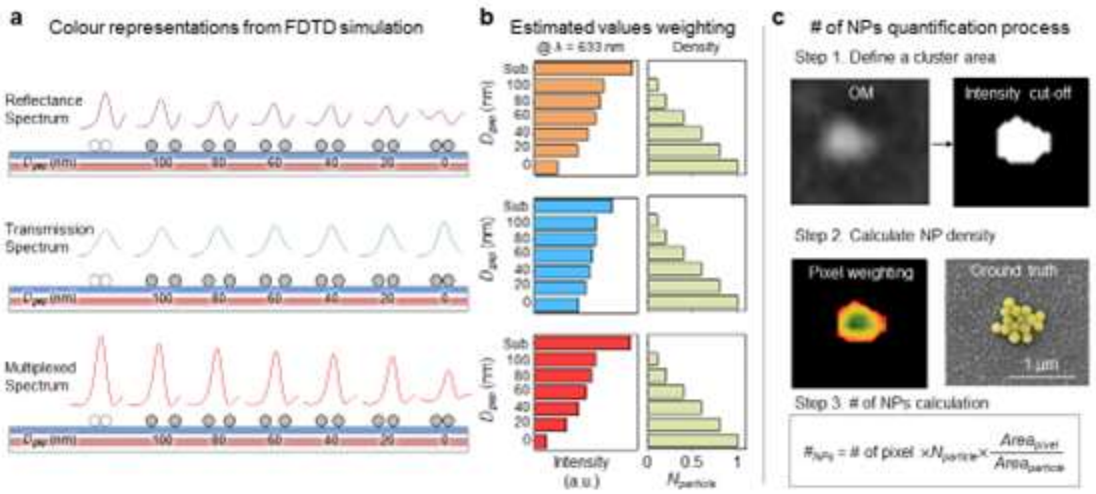

Figure S13. Quantification process with weighting factors. a) Schematic representation of the FDTD model with different gap distances between NPs, showing the resulting transmitted, reflected, and multiplexed spectra. b) Relationship between the intensity at the sensing wavelength (633 nm) and the corresponding matched density of NPs. c) Quantification process for determining the number of NPs based on the intensity-density relationship.

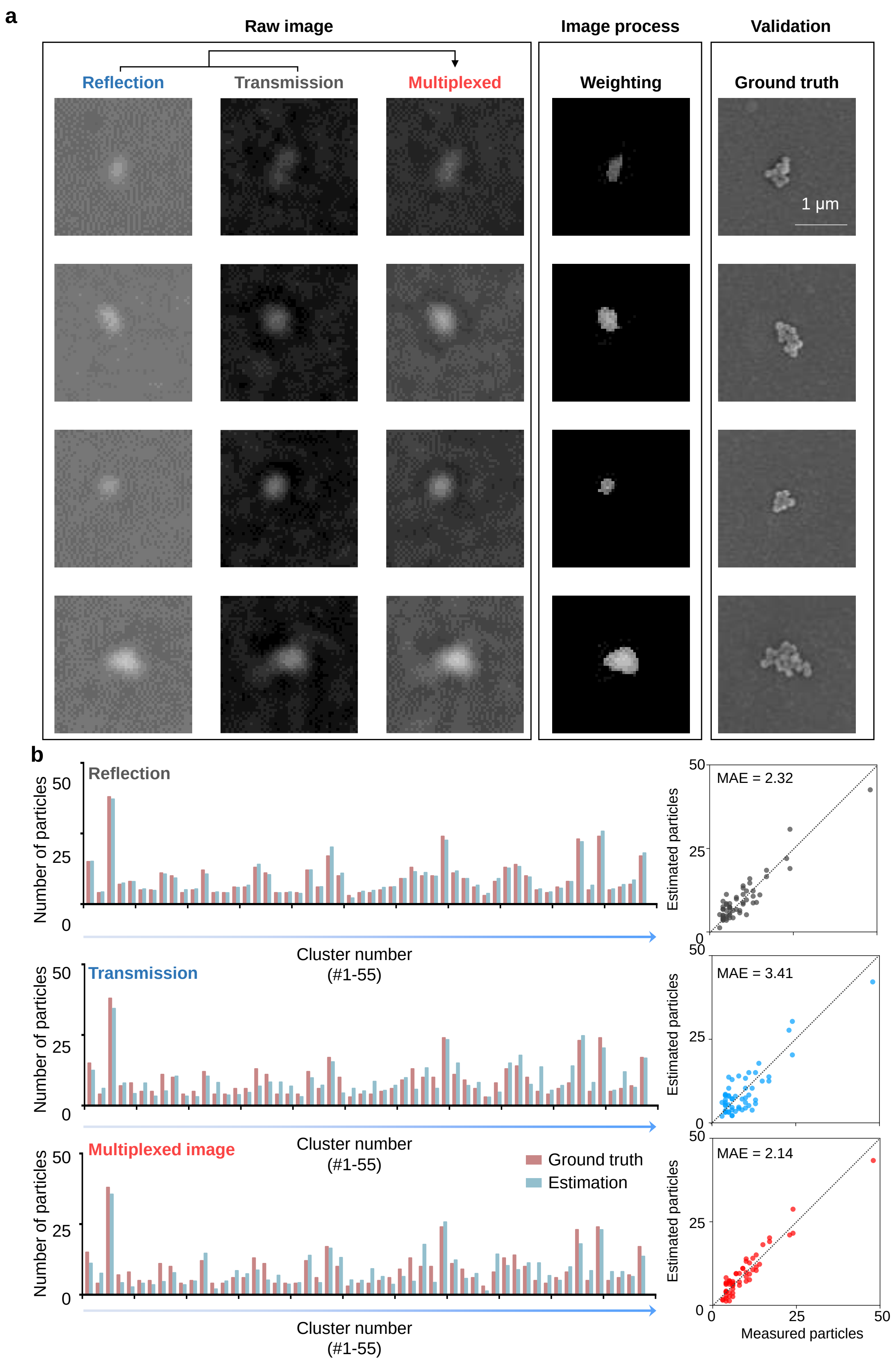
a
Raw image
Image process
Validation
Reflection
Transmission
Multiplexed
Weighting
Ground truth
1 μm
b
Reflection
Transmission
Multiplexed image
Number of particles
Cluster number
(#1-55)
Ground truth
Estimation
MAE = 2.32
MAE = 3.41
MAE = 2.14
Estimated particles
Measured particles

Figure S14. Cluster analysis of dual-imaging data and validation of quantification. a) Quantification of raw optical microscope images captured by dual-imaging system and ground truth validation with SEM image. b) Measured/estimated number of particles from reflectd, transmitted, and multiplexed image for different clusters and MAE.

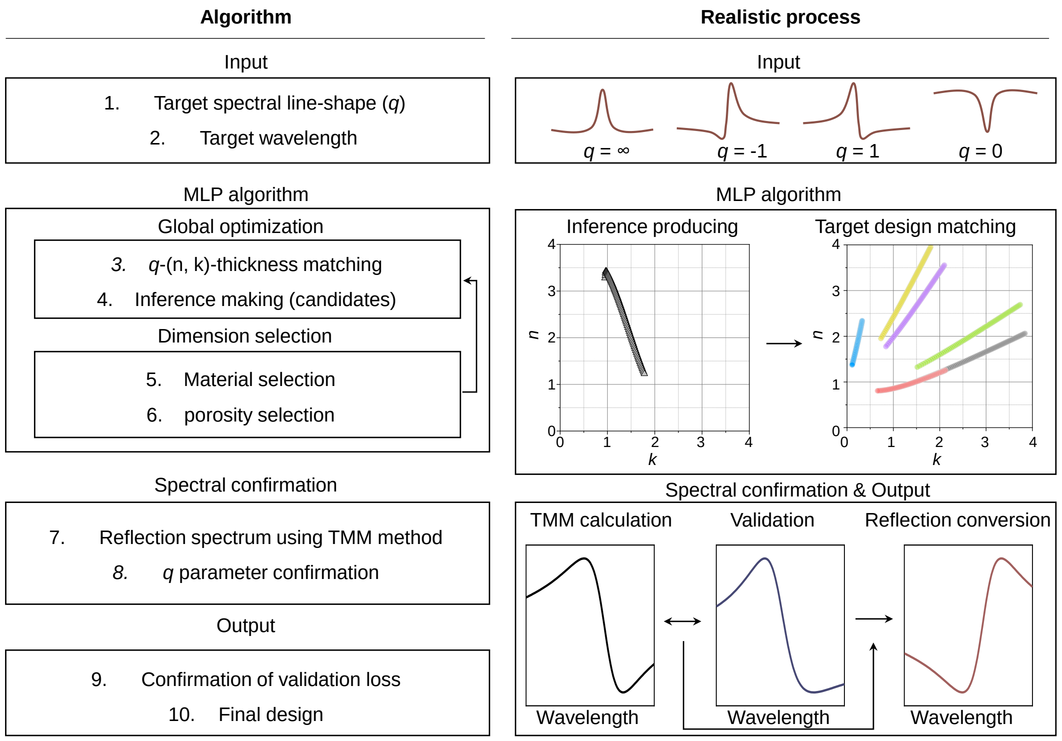

Figure S15. Overall algorithm from the input of target spectral shape to the final design. The multilayer perceptron (MLP) algorithm shows the overall learning process, including the input parameters and validation (left). The right side shows sequential flow from the input parameters to inference matching with validation process. TMM: transfer matrix method.

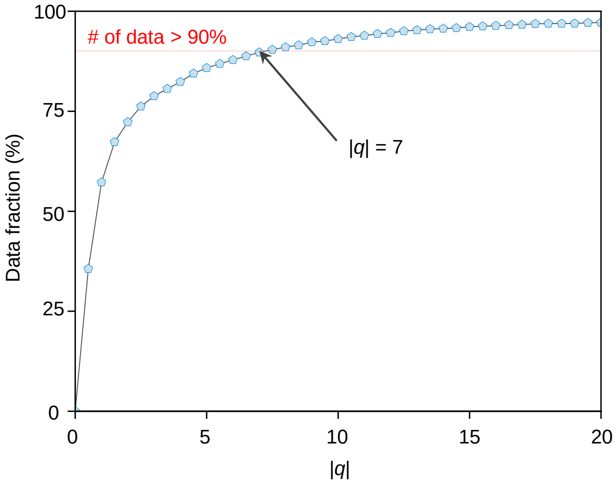

Figure S16. Data amount of parameter space. Data fraction from the total data set, which is expressed by the number of data set with smaller parameter *q* divided by the number of total data sets). This range includes 90% of the data set of Figure S1.

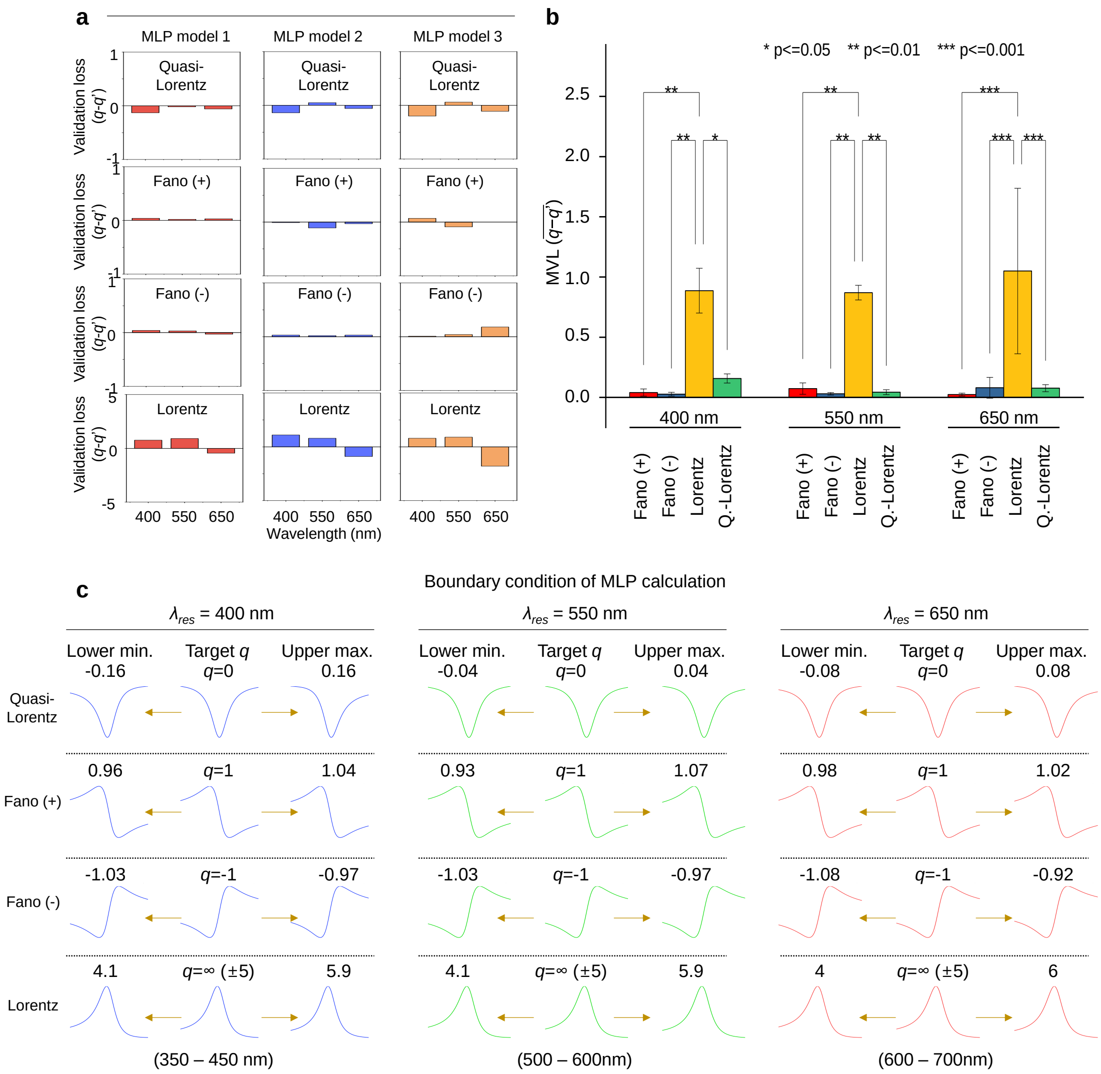

Figure S17. a) Validation loss of three individual MLP models for target wavelengths (400, 550, and 650 nm). b) Mean ± SD of the absolute value of (a), $n = 3$, $*p < 0.05$, $**p < 0.01$, and $***p < 0.001$. c) Effective range and boundary conditions of Fano spectral line shape from the target q parameter.

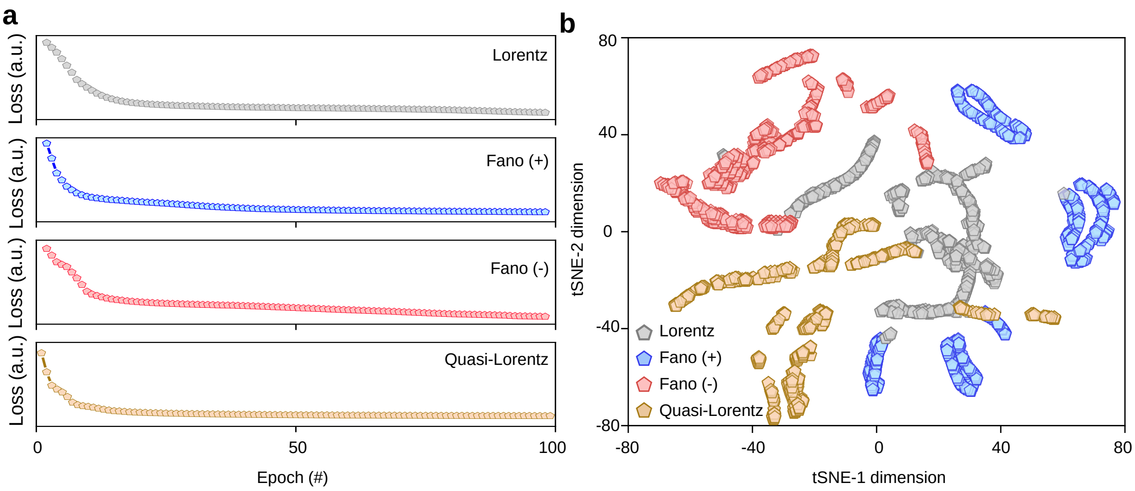

Figure S18. Spectral shape finding and evaluation of MLP model. a) Training losses from epochs of machine learning with target designs at λ_{res} = 400 nm. b) Visualization of sampled data set on two-dimensional (2D) space using t-SNE with perplexity = 30 and number of iterations = 1000. For each shape (Lorentz, Fano (+/−), and quasi-Lorentz), we randomly choose 1,000 cases and visualize them on 2D space.

Supplementary Table S1 | Calculations results of EE2 with target material and design parameters.

	Target wavelength = 400 nm	Target wavelength = 550 nm	Target wavelength = 650 nm
	Lorentz	Lorentz	Lorentz
Material	a-Si	a-Si	a-Ge
Porosity	35	15	25
Thickness	65	70	55
q-*q*'	2.24	1.6	2.13
	Fano (+)	Fano (+)	Fano (+)
Material	a-Si	a-Ge	a-GST
Porosity	0	70	20
Thickness	45	60	40
q-*q*'	0.1	0.33	0.42
	Fano (−)	Fano (−)	Fano (−)
Material	a-Ge	TiN	Co
Porosity	65	60	70
Thickness	10	60	4
q-*q*'	−0.25	−0.37	−0.69
	Quasi-Lorentz	Quasi-Lorentz	Quasi-Lorentz
Material	a-Si	c-GST	Co
Porosity	80	15	70
Thickness	60	45	60
q-*q*'	−0.58	−0.25	0.31

Supplementary Table S2 | Calculation results of MLP-based designer, including inference, target material and design parameters.

	Target wavelength = 400 nm		Target wavelength = 550 nm		Target wavelength = 650 nm	
Inference (n, k)	Lorentz		Lorentz		Lorentz	
	3.08	2.48	2.34	0.33	1.37	0.11
Material	a-Ge		a-Si		a-Si	
Porosity	0		0		80	
Thickness	49		70		20	
q'	0.76		0.91		-0.45	
Training loss	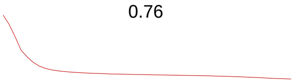 Epoch (0 - 100)		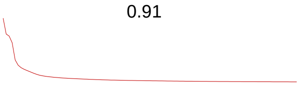 Epoch (0 - 100)		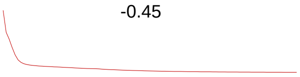 Epoch (0 - 100)	
Inference (n, k)	Fano (+)		Fano (+)		Fano (+)	
	1.79	1.24	1.90	0.24	2.28	0.13
Material	a-Ge		a-Si		a-Si	
Porosity	71		41		16	
Thickness	69		67		70	
q - q'	0.04		0.03		0.03	
Training loss	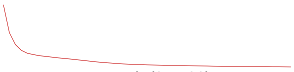 Epoch (0 - 100)		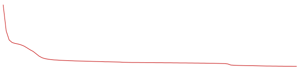 Epoch (0 - 100)		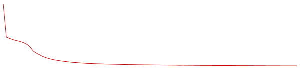 Epoch (0 - 100)	
Inference (n, k)	Fano (-)		Fano (-)		Fano (-)	
	1.36	2.43	0.81	0.76	1.93	3.59
Material	c_GST		TiN		Co	
Porosity	27		77		27	
Thickness	21		48		15	
q - q'	0.04		0.03		-0.03	
Training loss	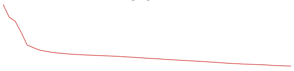 Epoch (0 - 100)		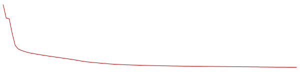 Epoch (0 - 100)		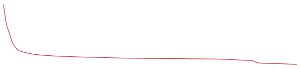 Epoch (0 - 100)	
Inference (n, k)	Quasi-Lorentz		Quasi-Lorentz		Quasi-Lorentz	
	0.87	1.13	1.64	2.96	1.69	1.67
Material	Co		Co		c-GST	
Porosity	79		38		80	
Thickness	67		51		67	
q - q'	-0.13		-0.02		-0.06	
Training loss	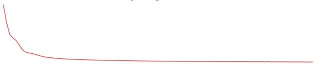 Epoch (0 - 100)		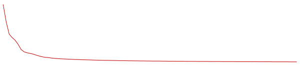 Epoch (0 - 100)		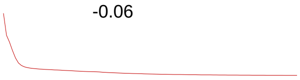 Epoch (0 - 100)	

References

[1] M. F. Limonov, M. V. Rybin, A. N. Poddubny, Y. S. Kivshar, *Nat. Photonics* **2017**, 11, 543.

[2] M. ElKabbash, T. Letsou, S. A. Jalil, N. Hoffman, J. Zhang, J. Rutledge, A. R. Lininger, C.-H. Fann, M. Hinczewski, G. Strangi, *Nat. Nanotechnol.* **2021**, 16, 440.

[3] A. Garahan, L. Pilon, J. Yin, I. Saxena, *J. Appl. Phys.* **2007**, 101, 014320.

[4] P. Johnson, R. Christy, *Phys. Rev. B* **1974**, 9, 5056.

[5] V. Schnabel, R. Spolenak, M. Doebeli, H. Galinski, *Adv. Opt. Mater.* **2018**, 6, 1800656.

[6] Y. Jiang, S. Pillai, M. A. Green, *Sci. Rep.* **2016**, 6, 30605.

[7] L. V. Rodríguez-de Marcos, J. I. Larruquert, J. A. Méndez, J. A. Aznárez, *Opt. Mater. Express* **2016**, 6, 3622.
